# Supplementary material for: Ripk3 signaling regulates HSCs during stress and represses radiation-induced leukemia in mice
Source: Stem Cell Reports. 2022 May 12;17(6):1428–41. doi: 10.1016/j.stemcr.2022.04.009 (PMC9213819; doi:10.1016/j.stemcr.2022.04.009)
Supplement: Document S1. Supplemental experimental procedures, Figures S1–S7, and Tables S1–S3 [file mmc1.pdf]

Stem Cell Reports, Volume *17*

Supplemental Information

Ripk3 signaling regulates HSCs during stress and represses radiation-induced leukemia in mice

Lei Zhang, Huacheng Luo, Hong-Min Ni, Shanhui Liu, Hongyun Xing, Jun Zhang, Mark Sellin, Peter Breslin, S.J., Wei Wei, Mitchell F. Denning, William Small Jr., Wen-Xing Ding, Suming Huang, and Jiwang Zhang

Supplemental figures

***Figure S1. Strategy for analysis of BM HSCs and HPCs*.**

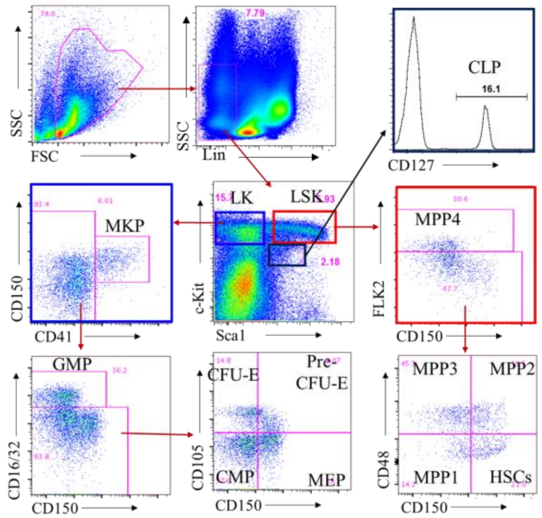

LSK populations:
$CD150^+FLk2^-CD48^-$ HSCs,
$CD150^-FLk2^-CD48^-$ MPP1,
$CD150^+FLk2^-CD48^+$ MPP2,
$CD150^-FLk2^-CD48^+$ MPP3
$CD150^-FLk2^+CD48^+$ MPP4.

LK-myeloid progenitors (LK-MPs):
$CD34^+CD16/32^-CD150^-CD105^-$ common myeloid progenitors (CMP),
$CD16/32^+$ granulocyte/monocyte progenitors (GMP),
$CD16/32^-CD150^+CD105^-$ megakaryocyte/erythrocyte progenitors (MEP),
$CD150^+CD41^+$ megakaryocyte progenitors (MKP),
$CD16/32^-CD150^+CD105^+$ pre-colony forming unit-erythroid (pre-CFU-E),
$CD16/32^-CD150^-CD105^+$ colony forming unit-erythroid (CFU-E)

$Lin^-Sca1^{low}c\text{-}kit^{low}CD127^+$ common lymphocyte progenitors (CLP)

Figure S2. Normal BM hematopoiesis in Ripk3$^{-/-}$ and Mlkl$^{-/-}$ mice under normal homeostatic conditions. (Associated with Figure 1) BM were collected from *WT*, *Ripk3*$^{-/-}$ and *Mlkl*$^{-/-}$ mice. Six mice per genotype were studied (3 males, 3 females). All mice were analyzed at 6 months of age. Percentages of LK, LSK, HSCs and MPPs (**a**), and percentages of MKP, CMP, GMP, MEP, pre-CFU-E, CFU-E, and CLP (**b**) in the BM of *WT*, *Ripk3*$^{-/-}$ and *Mlkl*$^{-/-}$ mice are shown.

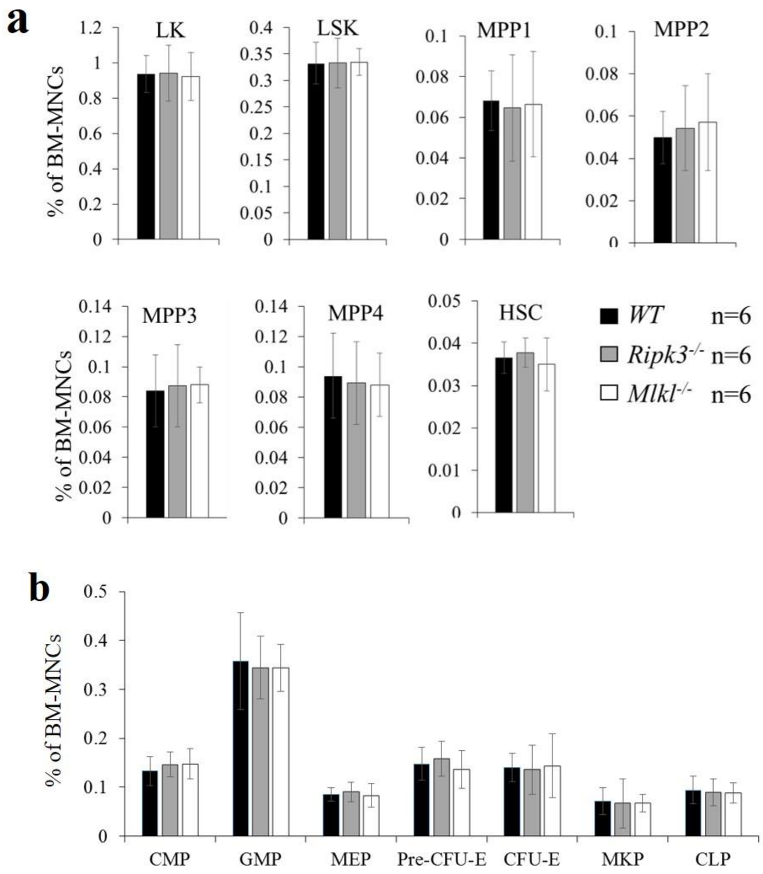

Figure S3. Ripk3 selectively regulates number and function of HSCs during serial transplantation in Mlkl-dependent and -independent manners, respectively. (Associated with Figure 2) **a-c**. BM MNCs were collected from 3rd transplantation recipients of the indicated genotypes of donors. Percentages of LK cells and LSK cells (**a**), as well as HSCs and MPPs (**b**) in BM from 3rd transplantation recipients of the indicated genotypes of donors are shown. The percentages of CD11b$^+$ granulocytes/monocytes, B220$^+$ B lymphocytes and CD3$^+$ T lymphocytes in PB were analyzed by flow cytometry (**c**). **d**. BM MNCs were collected from *WT*, *Ripk3*$^{-/-}$ and *Mlkl*$^{-/-}$ mice (all were CD45.2$^+$) and mixed with equal numbers of competitor BM MNCs (CD45.1$^+$), respectively. The mixture of cells was studied using serial transplantation assay. The CHRC of the donor BM MNCs was examined by analyzing the CD45.2$^+$ cell % in the PB of the recipients after 4 months of each cycle of transplantation. * and & indicate $p<0.05$ when compared to *WT* and *Ripk3*$^{-/-}$ groups, respectively.

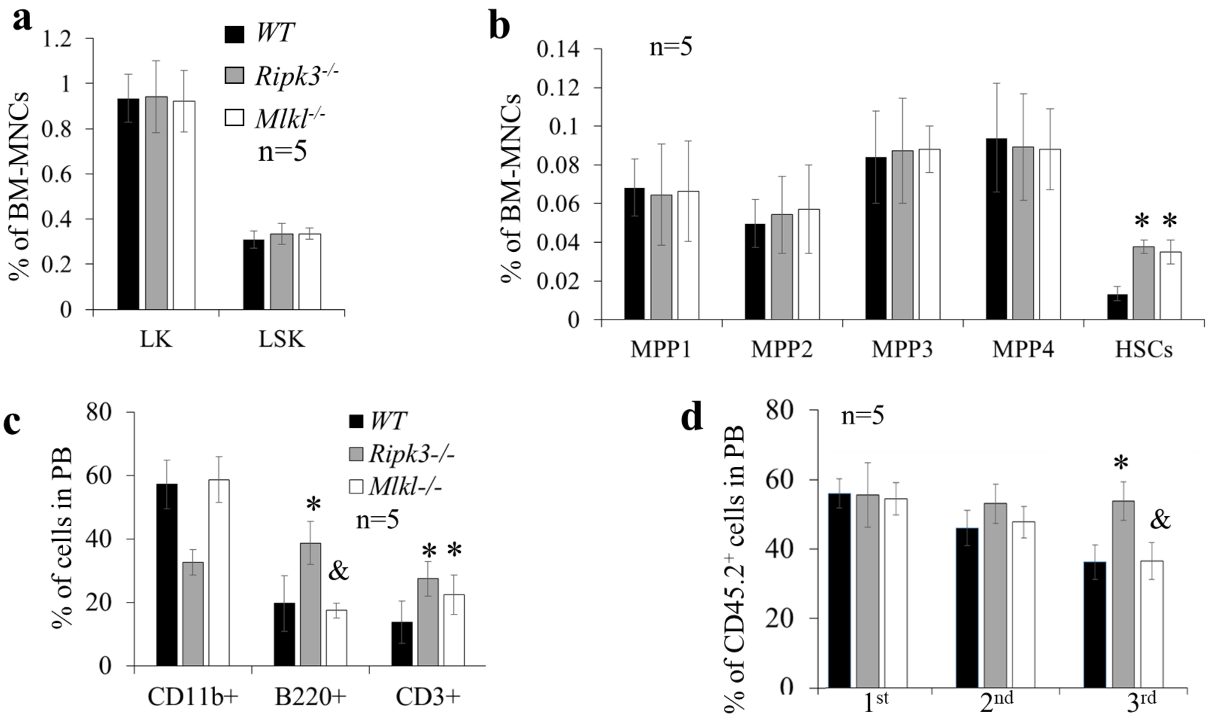

Figure S4. Ripk3 depletion prevents low dose radiation-induced loss of HSCs. (Associated with Figure 4) **a-b.** *WT, Ripk3*$^{-/-}$ and *Mlkl*$^{-/-}$ mice were irradiated with X-rays, 1.75Gy weekly × 4. BM MNCs were collected from mice 1 month following the last IR. Percentages of HSCs and HPCs (**a**) and the numbers of MNCs (**b**) in the BM of indicated genotypes of mice are presented. Five mice were studied in each group. * and ** stand for $p<0.05$ and $p<0.01$, respectively, when compared to non-irradiated *WT* mice. $ indicates $p<0.05$ when compared irradiated *Ripk3*$^{-/-}$ and *Mlkl*$^{-/-}$ mice. **c.** *WT* and *Ripk3*$^{-/-}$ mice were irradiated with 1.75Gy X-rays every week. BM MNCs were collected from mice 14 and 21 days following the first IR. HSCs and HPCs were analyzed by flow cytometry. Representative flow cytometric data are presented. & indicates $p<0.05$ when compared to irradiated *Ripk3*$^{-/-}$ mice. **d.** Lethally-irradiated mice (CD45.1$^+$) were transplanted with a mixture of *WT* (CD45.1$^+$) and *Ripk3*$^{-/-}$ (CD45.2$^+$) BM MNCs at a 1:1 ratio. One month after transplantation, mice were irradiated with 1.75Gy X-rays every week ×4 weeks. BM MNCs were collected 1 week following the last IR. The numbers of *WT* and *Ripk3*$^{-/-}$ HSCs and MPPs were analyzed by flow cytometry based on CD45.1 and CD45.2 expression. ** indicates $p<0.01$ compared to *WT* mice. **e.** *WT* mice were irradiated with 1.75Gy X-rays weekly. BM MNCs were collected from mice 14 and 30 days following the first IR. LSK-CD150$^+$CD48$^-$HSCs were purified by FACS and p-Ripk3 levels were analyzed by intracellular antibody staining. Data show one of the 3 biological triplicate experiments.

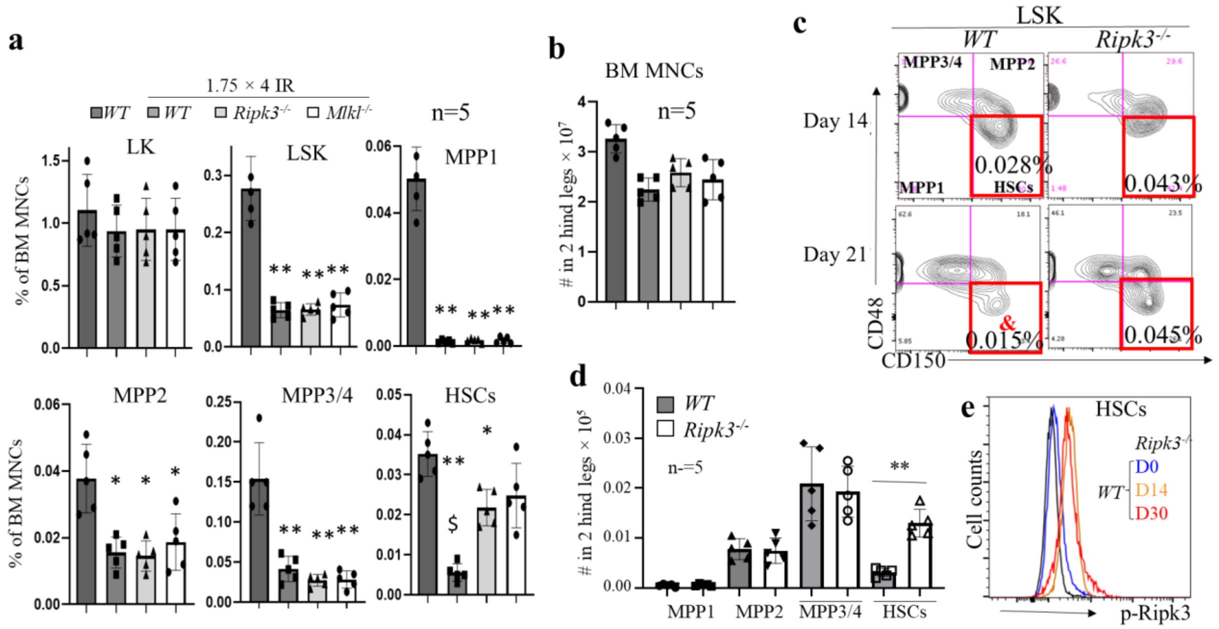

Figure S5. Depletion of either Tnfr or Ripk3 prevents low dose radiation-induced loss of HSCs. (Associated with Figure 4) **a-b.** *WT*, *Tnfr*$^{-/-}$ and *Ripk3*$^{-/-}$ mice were irradiated with 1.75Gy X-rays every week × 4 weeks. BM MNCs were collected 1 month following the last IR. p-Ripk3 levels (**a**) in LSK cells and numbers of HSCs (**b**) were analyzed by flow cytometry. Data in **a** show one of the 3 biological triplicate experiments. ** indicates for p<0.01 compared to *WT* mice.

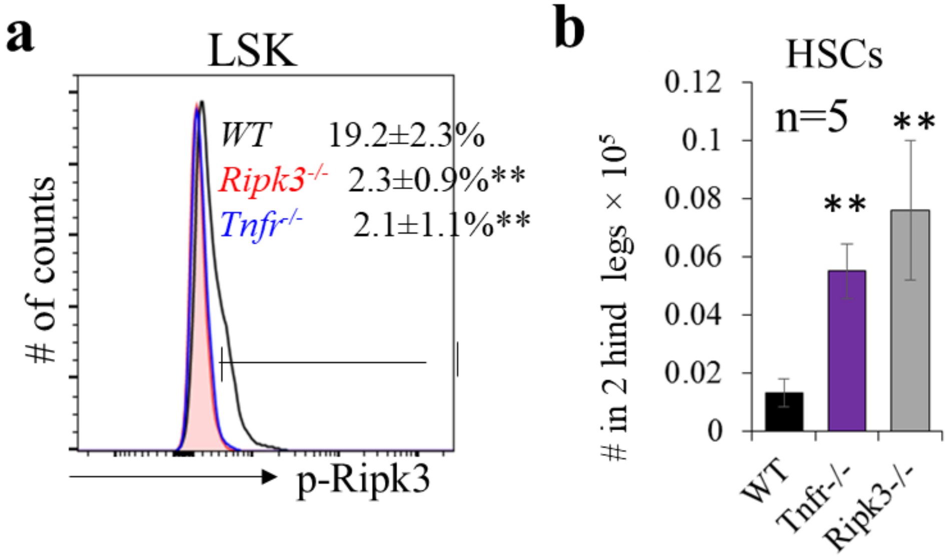

Figure S6. Tnf-Ripk3 signaling did not alter mitochondrial-ISR in LK HPCs. (Associated with Figure 6) *WT, Ripk3*$^{-/-}$, *Mlkl*$^{-/-}$ and *Tnfr*$^{-/-}$ mice were irradiated with X-ray, 1.75Gy weekly × 4. LK populations were collected from mouse BM 1 month after the last IR. p-eIF2a levels were examined by flow cytometry. Data in **a** and **c** show one of the 3 biological triplicate experiments.

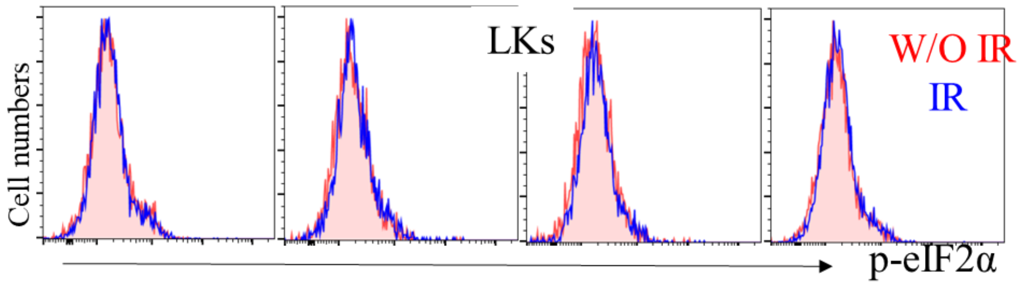

Figure S7. Inhibition of PDH or ROS restores eIF2α-Atf4-ISR signaling in radiated Mlkl$^{-/-}$ HSCs. (Associated with Figure 7) *Mlkl$^{-/-}$* mice were irradiated with X-ray, 1.75Gy weekly × 4 and treated with vehicle (Veh), CPI-613 or NAC, respectively. LSK cells were collected from mouse BM 1 month after the last IR. LSKs isolated from IR *Ripk3$^{-/-}$* mice were studied as controls. mtROS (**a**), p-eIF2a (**b**), protein synthesis rate (**c**), and senescence (**d**) were examined by flow cytometry. Data in **a-d** show one of the 3 biological triplicate experiments.

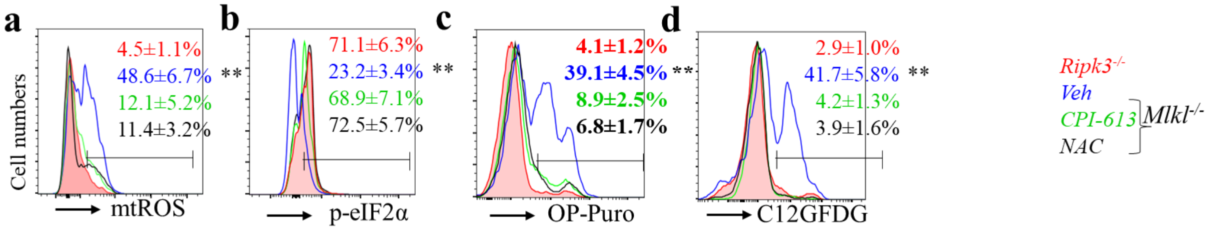

Table 1. Primers for mouse genotypic analysis:

PCR primer sequences for genotyping of *Ripk3*$^{-/-}$ mice
Ripk3-f: 5’-ggctttcattgtggaggtaagctgaga-3’;
Ripk3-r: 5’-gaacccgtggataagtgcacttgaat-3’.
These primers generate a 280bp product in *WT* and a 320bp product in *Ripk3*-mutant mice.
PCR primer sequences for genotyping of *Mlkl*$^{-/-}$ mice:
Mlkl-p1: 5’-tatgaccatggcaactcacg-3',
Mlkl-p2: 5'-accatctccccaaactgtga-3'
Mlkl-p3: 5'-tccttccagcacctcgta at-3'.
These primers generate a 498bp *WT* product and a 158bp product for the recombined Δ-exon 3 *Mlkl* gene.
PCR primer sequences for genotyping of *Tnfr*$^{-/-}$ mice:
Tnfr1a-p1: 5'-ggattgtcacggtgccgttgaag-3'
Tnfr1a-p2: 5'-tgacaaggacacggtgtgtggc-3'
Tnfr1a-p3: 5'-tgctgatggggatacatccatc-3'
These primers generate a 120bp *WT* product and a 155bp product in *Tnfr1a*-mutant mice.

Table 2. Primers used for qRT-PCR:

Taqman primers and probes	Purchased from Thermo Fisher Scientific
Bcl2	*Mm00477631_m1*
cIAP2	*Mm01168413_m1*
Bcl-x	*Mm00437783_m1*
Puma	*Mm00519268_m1*
NoxA	*Mm00451763_m1*
Bim	*Mm00437783_m1*
p16	*Mm00494449_m1*
p19	*Mm00486943_m1*
p15	*Mm00483241_m1*
p53	*Mm01731290_g1*
Gapdh	*Mm99999915_g1*
Calcrl	*Mm00516985_m1*
Klf4	*Mm00516104_m1*
Wbp4	*Mm01324202_m1*
Fkbp1a	*Mm01243847_g1*
P2ry13	*Mm00546978_m1*
Banp	*Mm00479456_m1*
Lcn2	*Mm01324470_m1*
Snx20	*Mm00446029_m1*
Primers	for qRT-PCR using SYBR Green assay
Atf4-f	5′-aacaagacagcagccactaggt,
Atf4-r	5′-tctgccttctctttcagagcctca
Grp78-f	5′-cagatcttctccacggct-3′
Grp78-r	5′-tgtcactcggagaatacc at-3′
Chop-f	5′-ccagaataacagccggaacctga-3′
Chop-r	5′-tcctgcagatcctcataccag-3′;
Erdj4-f	5′-ggatggttctagtagacaaagg-3′
Erdj4-r	5′-cttcgttgagtgacagtcctgc-3′
Dnajb8-f	5′-tcggtgctggtcatcccttt-3′
Dnajb8-r	5′-gggaactcaccaaagcccgaa-3′
Xbp1s-f	5′-gagtccgcagcaggtg-3′
Xbp1s-r	5′-gtgtcagagtccatggga-3′

Xbp1 total-f	5′-aaacagagtagcagcgcagac-3′
Xbp1 total-r	5′-caggatccagcgtgtcca t-3′
β-actin-f	5′-ggctgtattcccctccatcg-3′
β-actin-r	5′-ccagttggtaacaatgccatg t-3′
RT-PCR primers	for detecting xbp1 splicing:
xbp1 splicing-f	5′-acacgcttgggaatggaac-3′
xbp1 splicing-r	5′-ccatgggaagatgttctggg-3′

Table 3. Antibodies for flow cytometric analysis and Western blot analysis

**Antibodies for intracellular staining and flow cytometry**	Venders	Cat #
Phospho-p38 MAPK (Thr180/Tyr182) (3D7) Rabbit mAb (Alexa Fluor® 647 Conjugate)	Cell Signaling Technology	14594
Phospho-NF-κB p65 (Ser536) (93H1) Rabbit mAb (Alexa Fluor® 647 Conjugate)	Cell Signaling Technology	4887
Phospho-RIP3 (Thr231/Ser232) (E7S1R) Rabbit mAb (Alexa Fluor® 488 Conjugate)	Cell Signaling Technology	47477
p44/42 MAPK (Erk1/2) (137F5) Rabbit mAb (Alexa Fluor® 647 Conjugate)	Cell Signaling Technology	5376
Phospho-GSK-3β (Ser9) (D85E12) XP® Rabbit mAb (Alexa Fluor® 647 Conjugate)	Cell Signaling Technology	14332
Phospho-eIF2α (Ser51) (D9G8) XP® Rabbit mAb (Biotinylated)	Cell Signaling Technology	5199
Alexa Fluor 488 Goat anti-Rabbit IgG	ThermoFisher Scientific	A-11034
Rabbit monoclonal phospho-eIF2a (Ser51)	ThermoFisher Scientific	44-728G
FAM-FLICA® Caspase-1 (YVAD) Assay Kit	ImmunoChemistry Technologies	97
**Antibodies for Western blotting**		
RIP3 (D4G2A) Rabbit mAb (Mouse-Specific)	Cell Signaling Technology	95702
Anti-RIP3 antibody (Mouse-Specific)	Abcam	ab56164
Phospho-RIP3 (Thr231/Ser232) Antibody (Mouse-Specific)	Cell Signaling Technology	57220
Phospho-MLKL (Ser345) (D6E3G) Rabbit mAb	Cell Signaling Technology	37333
MLKL (D6W1K) Rabbit mAb (Mouse-Specific)	Cell Signaling Technology	37705
OPA1 (D6U6N) Rabbit mAb	Cell Signaling Technology	80471
ATF-4 (D4B8) Rabbit mAb	Cell Signaling Technology	11815
Phospho-PERK (Thr980) Monoclonal Antibody (G.305.4)	Thermofisher Scientific	MA5-15033
**Antibodies for HSCs and HPCs analysis**		
Brilliant Violet 421™ anti-mouse Ly-6G/Ly-6C (Gr-1) Antibody	Biolegend	108434
Brilliant Violet 421™ anti-mouse CD3ε Antibody	Biolegend	100336
Brilliant Violet 421™ anti-mouse B220 Antibody	Biolegend	103240
Brilliant Violet 421™ anti-mouse TER-119/Erythroid Cells Antibody	Biolegend	116233
PE anti-mouse Ly-6A/E (Sca-1) Antibody	Biolegend	108108
APC anti-mouse CD117 (c-Kit) Antibody	Biolegend	105812
APC/Cy7 anti-mouse CD48 Antibody	Biolegend	103431
PE/Cy7 anti-mouse CD150 (SLAM) Antibody	Biolegend	115914
PE/Cy5 anti-mouse CD135 Antibody	Biolegend	135312
PerCP/Cy5.5 anti-mouse CD127 (IL-7Rα) Antibody	Biolegend	135022
CD41a Monoclonal Antibody (eBioMWReg30 (MWReg30)), PerCP-eFluor 710	Life technologies	46-0411
FITC anti-mouse CD117	BD Bioscience	553354
PE-Cy7 anti-mouse CD3e	BD Bioscience	552774
PE anti-mouse CD11b	Thermo Fisher Scientific	12-0112-85
APC Anti-human/mouse CD45R	Thermo Fisher Scientific	17-0452-82
PE anti-mouse CD8a	Thermo Fisher Scientific	12-0081-82
APC Anti-mouse CD4	Thermo Fisher Scientific	17-0041-83
FITC CD3e Monoclonal Antibody (145-2C11),	Thermo Fisher Scientific	11-0031-82

Supplementary Material and Methods

Analysis of levels of intracellular ROS and mitochondrial ROS. BM-MNCs (1×10^6/ml) were stained with antibodies against lineage markers, anti-Sca-1-PE/FITC and anti-c-kit-APC antibodies in PBS supplemented with 5mM glucose, 1mM $CaCl_2$, 0.5mM $MgSO_4$, and 5mg/ml BSA and then incubated with 10µM of the cell-permeant 2',7'-dichlorodihydrofluorescein diacetate (H2DCFDA) (ThermoFisher, D399) or MitoSOX™ Red Mitochondrial Superoxide Indicator (ThermoFisher, M36008) for 10-30 min. at 37 ℃. Levels of intracellular ROS and mitochondrial ROS in LK- and LSK-cells were analyzed using a FACS Fortessa flow cytometer (Becton-Dickinson, San Jose, CA, USA). For each sample, a minimum of 300,000 BM-MNCs were acquired and the data were analyzed using Flowjo software. In all experiments, PE and APC isotype controls and other positive and negative controls were included as appropriate.

MitoTracker analysis: Bone marrow cells were incubated in 20nM MitoTracker Green (M7514; Molecular Probes) according to the manufacturer's instructions for 15 min. at 37 ℃. After washing the cells twice with PBS, cells were stained with HSC cell surface markers. Cells were washed twice with PBS and then analyzed using FACS.

Senescence analysis. SA-β-gal activity in sorted LSK cells was determined using a Senescence β-Galactosidase Staining Kit from Cell Signaling Technology (#9860) according to the manufacturer's instructions and analyzed by flow cytometry.

Seahorse Assays. XF96 extracellular flux assay kits (Agilent Technologies, 102417-100) were used to measure oxygen consumption (OCR). LSK cells were sorted, and directly plated into XF96 cell culture microplates. OCR was measured according to the protocol supplied by the manufacturer. Briefly, five replicate wells of 1-2 $\times 10^5$ cells were seeded into a Cell-Tak coated 96-well XF96 well plate (Corning, 324240). Thirty minutes prior to analysis, the medium was replaced with Seahorse XF medium (Agilent Technologies, 102353-100) and the plate was incubated at 37°C. Analyses were performed at basal conditions.

Measuring protein synthesis. BM MNCs were collected from mice 1 hour after intraperitoneal administration of OP-Puro 50mg/kg body weight (Opropargyl-puromycin; pH 6.4-6.6 in PBS). BM MNCs (4×10^6) were stained with antibodies against cell surface markers as described above. After washing, cells were fixed, permeabilized, and the azide-alkyne cycloaddition was performed using the Click-iT Cell Reaction Buffer Kit (Life Technologies, Cat#: C10269) with 5µM Alexa Fluor™ 488 Azide (Life Technologies). The protein synthesis rate was measured by flow cytometry.

Analysis of apoptosis using Annexin-V staining and caspase activity. LK and LSK cells were stained with propidium iodide and APC-conjugated Annexin-V in Annexin binding buffer following the manufacturer's instructions (BD Biosciences). Apoptosis was further verified by examining Caspase 3 activity using active-Caspase-3 staining kits following the manufacturer's instructions (eBioscience).

Colony-forming cell (CFC) assay. The CFC assay was performed by culturing BM-MNCs in MethoCult GF M3434 methylcellulose medium (Stem Cell Technologies, Vancouver, BC). Colonies of CFU-granulocyte macrophage (GM) and burst-forming unit-erythroid (BFU-E) were

scored on day 7 and those of CFU-granulocyte, -erythrocyte, -monocyte, and -megakaryocyte (GEMM) were scored on day 12 of incubation according to the manufacturer's protocol.

Western blotting. Cell lysates from BM MNCs were extracted using Cell Lysis Buffer (Cell Signaling) followed by a brief sonication. The supernatants were collected after centrifugation at 4 ℃., 14,000 ×g for 20 min. Protein samples were separated by SDS-PAGE on 10% or 12% acrylamide gels. Proteins were transferred onto nitrocellulose membranes to examine the target proteins by antibody blotting. Antibodies used in this study are listed in Antibodies for Western blotting (**Table 3**). The band quantifications using Multi Gauge 3.0 were normalized to corresponding controls, which were set to 1.00 after being normalized to corresponding loading standards on the Western blot.

Serial transplantation assay. BM-MNCs were collected from 2 month-old *WT*, *Ripk3*$^{-/-}$ and *Mlkl*$^{-/-}$ mice (CD45.2^{+}) and were transplanted into lethally-irradiated (9.5Gy IR) Ptprc (CD45.1^{+}) mice. Five recipients were used in each group, 5×10^{6} MNCs were transplanted per recipient. PB and BM were collected from recipient mice 4 months post-transplantation for phenotypic analysis using flow cytometry. MNCs from all 5 recipients in each group were pooled and transplanted into lethally-irradiated 2nd Ptprc recipients, respectively. Five recipients were used in each group, 1×10^{7} MNCs were transplanted per recipient. The same procedure was followed for the 3rd transplantation.

Competitive repopulation and serial transplantation assay. Donor BM-MNCs were collected from 2 month-old *WT, Ripk3*$^{-/-}$ and *Mlkl*$^{-/-}$ mice (CD45.2^{+}) and were mixed, respectively, with equal numbers of competitor BM-NMCs collected from 2-month-old Ptprc (CD45.1^{+}) mice. The mixtures of BM cells were transplanted into lethally-irradiated (9.5Gy) Ptprc (CD45.1^{+}) mice by tail-vein injection. Five recipient mice were used in each group, 5×10^{5} donor NMCs and 5×10^{5} competitor NMCs per recipient. PB samples were collected from all recipients 4 months after transplantation. Donor cell engraftments were analyzed by examining % of CD45.2^{+} cells in PB as assessed by flow cytometry. T-cell, B-cell, granulocyte, and monocyte/macrophage lineage contributions of the donor cells were analyzed by staining with anti-CD3, anti-B220, anti-Gr-1, and anti-Mac-1, respectively. For 2nd transplantations, MNCs from all 5 recipients in each group were pooled and transplanted into lethally-irradiated 2nd Ptprc recipients, respectively. Five recipients were used per group, 1×10^{7} MNCs per recipient. The exact same procedures were followed for the 3rd transplantation.

RNAseq Analysis. LSK and LK cells were purified from BM of *WT* and *Ripk3*$^{-/-}$ mice by FACS 1 month after 1.75×4 IR. Two biological replicates were analyzed for each sample. Each sample consisted of a mixture of the indicated cells from 4-5 mice. Total RNA was isolated from these cells using TRIzol reagent (Invitrogen, Carlsbad, CA) following the manufacturer's protocol. Raw data FASTQ files were aligned to the mouse genome (mm9) using Tophat (version 2.0) and Bowtie2.(Langmead et al., 2009) TopHat software was used to analyze RNA-Seq data in order to discover splice junctions.(Bai et al., 2016) Gene expression profiles for the individual samples were calculated as FPKM (paired-end fragments per kilobase of exon model per million mapped reads) values. The Gene Ontology analysis was carried out with the Database for Annotation, Visualization and Integrated Discovery (DAVID) tool (https://david.ncifcrf.gov/, Version 6.8).(Huang da et al., 2009) The differential expression cluster for heatmap was generated using

JavaTreeview.(Saldanha, 2004) The RNAseq dataset was deposited into the Gene Expression Omnibus archive (accession no. GSE70899) and GEO number (GSE139143).

Statistical analyses. Data are expressed as means ± SD. Two-way ANOVA (multiple groups) and Student's *t*-test (two groups) were performed to determine the statistical significance of differences among and between experimental groups. $P<0.05$ was considered significant. All these analyses were done using GraphPad Prism from GraphPad Software (San Diego, CA).

References

Bai, Y., Kinne, J., Donham, B., Jiang, F., Ding, L., Hassler, J.R., and Kaufman, R.J. (2016). Read-Split-Run: an improved bioinformatics pipeline for identification of genome-wide non-canonical spliced regions using RNA-Seq data. BMC genomics *17 Suppl 7*, 503.

Huang da, W., Sherman, B.T., and Lempicki, R.A. (2009). Systematic and integrative analysis of large gene lists using DAVID bioinformatics resources. Nature protocols *4*, 44-57.

Langmead, B., Trapnell, C., Pop, M., and Salzberg, S.L. (2009). Ultrafast and memory-efficient alignment of short DNA sequences to the human genome. Genome Biol *10*, R25.

Saldanha, A.J. (2004). Java Treeview--extensible visualization of microarray data. Bioinformatics *20*, 3246-3248.
