# Supplementary material for: Ripk3 signaling regulates HSCs during stress and represses radiation-induced leukemia in mice
Source: Stem Cell Reports. 2022 May 12;17(6):1428–41. doi: 10.1016/j.stemcr.2022.04.009 (PMC9213819; doi:10.1016/j.stemcr.2022.04.009)
Supplement: Document S2. Article plus supplemental information [file mmc2.pdf]

Stem Cell Reports
Article
OPEN ACCESS

Ripk3 signaling regulates HSCs during stress and represses radiation-induced leukemia in mice

Lei Zhang,[1,2,9] Huacheng Luo,[3,9] Hong-Min Ni,[4] Shanhui Liu,[1] Hongyun Xing,[1] Jun Zhang,[5] Mark Sellin,[1] Peter Breslin, S.J.,[1,6] Wei Wei,[1] Mitchell F. Denning,[1] William Small, Jr.,[7] Wen-Xing Ding,[4] Suming Huang,[3,*] and Jiwang Zhang[1,7,8,*]

[1]Department of Cancer Biology, Oncology Research Institute, Cardinal Bernardin Cancer Center, Loyola University Chicago, Maywood, IL 60153, USA
[2]Cyrus Tang Hematology Center, Collaborative Innovation Center of Hematology, National Clinical Research Center for Hematologic Diseases, Soochow University, Suzhou 215123, China
[3]Department of Pediatrics, Pennsylvania State University College of Medicine, Hershey, PA 17033, USA
[4]Department of Pharmacology, Toxicology and Therapeutics, University of Kansas Medical Center, Kansas City, KS 66160, USA
[5]Department of Biology, College of Life Sciences, Shanghai Normal University, Shanghai 200234, People's Republic of China
[6]Departments of Molecular/Cellular Physiology and Biology, Loyola University Chicago, Chicago, IL 60660, USA
[7]Department of Radiation Oncology, Loyola University Medical Center, Maywood, IL 60153, USA
[8]Department of Pathology, Loyola University Medical Center, Maywood, IL 60153, USA
[9]These authors contributed equally
*Correspondence: shuang4@pennstatehealth.psu.edu (S.H.), jzhang@luc.edu (J.Z.)
https://doi.org/10.1016/j.stemcr.2022.04.009

SUMMARY

Receptor-interacting protein kinase 3 (Ripk3) is one of the critical mediators of inflammatory cytokine-stimulated signaling. Here we show that Ripk3 signaling selectively regulates both the number and the function of hematopoietic stem cells (HSCs) during stress conditions. Ripk3 signaling is not required for normal homeostatic hematopoiesis. However, in response to serial transplantation, inactivation of Ripk3 signaling prevents stress-induced HSC exhaustion and functional HSC attenuation, while in response to fractionated low doses of ionizing radiation (IR), inactivation of Ripk3 signaling accelerates leukemia/lymphoma development. In both situations, Ripk3 signaling is primarily stimulated by tumor necrosis factor-α. Activated Ripk3 signaling promotes the elimination of HSCs during serial transplantation and pre-leukemia stem cells (pre-LSCs) during fractionated IR by inducing Mlkl-dependent necroptosis. Activated Ripk3 signaling also attenuates HSC functioning and represses a pre-LSC-to-LSC transformation by promoting Mlkl-independent senescence. Furthermore, we demonstrate that Ripk3 signaling induces senescence in HSCs and pre-LSCs by attenuating ISR-mediated mitochondrial quality control.

INTRODUCTION

Receptor-interacting protein kinase 3 (Ripk3) is a serine/threonine protein kinase that induces necroptosis, a type of programmed cellular necrosis, by phosphorylation activation of mixed-lineage kinase domain-like protein (Mlkl). In most types of tissue cells, Ripk3 activation is stimulated by tumor necrosis factor-α (Tnf-α) through induction of the formation of a Ripk1 and Ripk3 complex (a necroptosome) via receptor-interacting protein kinase homotypic interaction motif (RHIM) domain-mediated Ripk1 and Ripk3 interaction (Van Herreweghe et al., 2010). Ripk3 activation can also be stimulated by interferon (Ifn), interleukin-1β (IL-1β), toll-like receptor (Tlr) signaling, or viral infection by inducing the interaction of Ripk3 with other RHIM proteins, including DNA-dependent activator of Ifn-regulatory factors (Dai), also known as Zbp1, and Tir-domain-containing adapter-inducing Ifn-β (Trif), also known as Ticam1 (Brault et al., 2018; Kaiser et al., 2013; Schock et al., 2017; Upton et al., 2012). Activated Ripk3 phosphorylates Mlkl, which promotes oligomerization and plasma membrane localization of Mlkl, inducing membrane permeabilization and necroptosis. In addition, Ripk3 can also induce reactive oxygen species (ROS) production, probably by phosphorylating mitochondrial proteins such as Pdc-E3, Pygl, Glul, and Gludl, and cytokine production by stimulating the activation of NF-κB and inflammasomes (Kang et al., 2015; Moriwaki et al., 2014; Wang et al., 2014).

Studies have suggested that Ripk3 signaling plays critical roles in the pathogenesis of many hematopoietic disorders (Hockendorf et al., 2016; Wagner et al., 2019; Xin et al., 2016a, 2016b). Some leukemia-related mutations induce the activation of Ripk3 signaling in hematopoietic cells (Hockendorf et al., 2016; Lee et al., 2018). Ripk3/Mlkl-mediated necroptosis is detected in bone marrow (BM) samples from patients with aplastic anemia (AA) and myelodysplastic syndromes (MDS) (Wagner et al., 2019; Xin et al., 2016a). Inactivation of Ripk3 signaling prevents T cell activation and AA development in a *Tak1*-mutant mouse model (Xin et al., 2016a). In acute myeloid leukemia (AML), RIPK3 signaling might be epigenetically repressed in leukemic cells isolated from a subgroup of AML patients such as AML with *RUNX1-ETO* or *FLT3-ITD* mutations (Hockendorf et al., 2016). Ripk3 signaling represses the development of such sub-types of AML by stimulating Mlkl-mediated necroptosis and IL-1β-induced differentiation of pre-leukemia stem cells

 | © 2022 The Author(s).
This is an open access article under the CC BY license (http://creativecommons.org/licenses/by/4.0/).

(pre-LSCs) as demonstrated in mouse models (Hockendorf et al., 2016). However, in HOXA-expressing AML, such as AML with *NPM1* or *MLL* gene mutations, RIPK3 signaling is activated, which promotes such AML development by repressing the differentiation of leukemic cells via inhibition of JAK1-STAT1 signaling (Xin et al., 2016b). However, the role of Ripk3 signaling in the regulation of normal and stress hematopoiesis has not been studied.

Exposure to ionizing radiation (IR) induces both acute tissue damage and chronic long-term residual damage (LT-RD) in the body. Among all tissues, BM is the most sensitive to both IR-induced acute tissue damage and LT-RD. After IR, BM failure and reduction of peripheral blood (PB) cell counts often occur within days due to the massive death of hematopoietic stem cells (HSCs) and hematopoietic progenitor cells (HPCs). Compared with proliferative HPCs, quiescent HSCs are relatively resistant to IR-induced death. However, due to the quiescent cell-cycle status and lower fidelity of DNA repair in this instance, some HSCs cannot fully repair chromosomal damage and thus become senescent (a permanent cell-cycle-arrested status). Such residually damaged HSCs cause LT-RD in BM as demonstrated by abnormal dysplastic hematopoiesis in most IR victims, followed by increased risk for the development of leukemia (Carbonneau et al., 2012; Gutierrez-Martinez et al., 2018; Shao et al., 2010, 2014). Thus, IR exposure is an established cause of BM failure and leukemia.

In addition to directly inducing damage in DNA, IR also induces the generation of ROS and subsequent secondary reactive pro-inflammatory processes and innate immune responses (Cadet and Davies, 2017; Kim et al., 2014; Mavragani et al., 2016). All these are believed to contribute to both acute BM damage and LT-RD. However, the molecular mechanism by which IR induces acute BM damage, LT-RD, and leukemia pathogenesis has still not been fully elucidated (Wang et al., 2010). Most previous studies suggested that the acute BM damage is primarily mediated by IR-induced ROS production and DNA damage through the activation of p53-Puma-dependent apoptosis in proliferative HPCs, while LT-RD is mediated by long-lived free radicals and pro-inflammatory cytokines/chemokines, which induce mitochondrial damage and senescence in quiescent HSCs. The accumulation of additional genetic mutations and escape from senescence are the potential mechanisms underlying the development of cancer (Palacio et al., 2017).

By comparing hematopoiesis among wild-type (WT), *Ripk3*-knockout (*Ripk3*$^{-/-}$), Tnf-α receptor knockout (*Tnfr*$^{-/-}$), and *Mlk1*-knockout (*Mlkl*$^{-/-}$) mice under normal homeostatic conditions, serial transplantation-related stress conditions, and IR-stress conditions, we demonstrate here a specific role for Ripk3 signaling in the regulation of both the number and the function of HSCs in the stressed tissue setting.

RESULTS

Normal hematopoiesis in *Ripk3*$^{-/-}$ mice and *Mlkl*$^{-/-}$ mice during homeostasis

Ripk3$^{-/-}$ and *Mlkl*$^{-/-}$ mice were maintained in a healthy condition without any signs of disease to a minimum age of 1$^1/_2$ years in a germ-free barrier facility. White blood cell (WBC) counts, hemoglobin (Hb) concentration, platelet (Plt) numbers, and lymphocyte/myeloid cell ratio in PB were comparable among *Ripk3*$^{-/-}$ mice, *Mlkl*$^{-/-}$ mice, and their age/gender-matched WT mice (Figure 1). In the BM, the percentages and absolute numbers of Lin$^-$Sca1$^+$c-kit$^+$ (LSK) and Lin$^-$Sca1$^-$c-kit$^+$ (LK) populations were also comparable among WT, *Ripk3*$^{-/-}$, and *Mlkl*$^{-/-}$ mice. Furthermore, HSCs and multipotent progenitor 1 (MPP1), MPP2, MPP3, and MPP4 cells within the LSK population, and common myeloid progenitors (CMPs), granulocyte/monocyte progenitors (GMPs), megakaryocyte/erythrocyte progenitors (MEPs), megakaryocyte progenitors (MKPs), and pre-colony forming unit-erythroid (pre-CFU-E) and CFU-E cells within the LK-myeloid progenitors (LK-MPs), as well as common lymphocyte progenitors (CLPs), were also comparable among WT, *Ripk3*$^{-/-}$, and *Mlkl*$^{-/-}$ mice (Figures 1, S1, and S2).

Inactivation of Ripk3 signaling prevents Mlkl-mediated HSC exhaustion and ROS-p38-mediated senescence during serial transplantation

Ripk3-Mlkl signaling was not activated in HSCs under homeostatic conditions but was induced during serial transplantation in BM mononucleated cells (MNCs) as shown by western blotting and in LSK-HSPCs (HSCs + MPPs) as demonstrated by flow cytometric assay (Figures 2A–2C). To study the role of Ripk3-Mlkl signaling in the regulation of HSC self-renewal, we first conducted serial transplantation experiments without using competitor BM cells. BM and PB of the recipient mice were analyzed 4 months after each round of transplantation. We found that the percentages and absolute numbers of HSCs, MPPs, and LK cells (including CMP, GMP, MEP, MKP, pre-CFU-E, and CFU-E) were comparable among mice that had received *Ripk3*$^{-/-}$, *Mlkl*$^{-/-}$, and WT BM cells in the first and second transplantations (Figure 2D and data not shown). However, during the third transplantation, although the percentages and absolute numbers of MPPs and LK cells were still comparable in all recipient mice regardless of the genotype of BM cells transplanted, the numbers of HSCs were significantly reduced in mice that had received WT BM cells compared with mice receiving either *Ripk3*$^{-/-}$ or *Mlkl*$^{-/-}$ BM cells (Figures 2D–2F, S3A, and S3B). These data suggest that inactivation of Ripk3-Mlkl signaling selectively prevents the loss of HSCs during serial transplantation. Interestingly, when *Ripk3*$^{-/-}$, *Mlkl*$^{-/-}$, and WT BM cells were collected

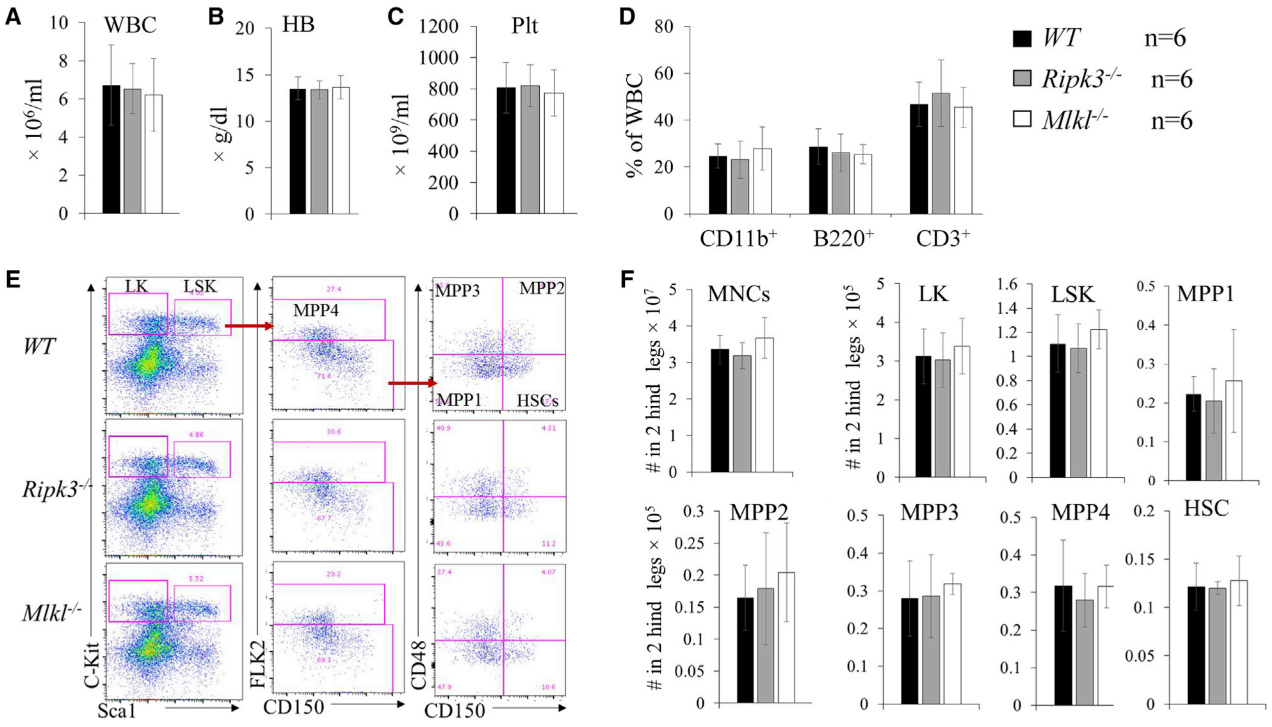

Figure 1. Normal hematopoiesis in Ripk3$^{-/-}$ and Mlkl$^{-/-}$ mice under normal homeostatic conditions

(A–F) PB (A–D) and BM (E and F) were collected from WT, *Ripk3*$^{-/-}$, and *Mlkl*$^{-/-}$ mice. Six mice per genotype were studied (three males and three females). All mice were analyzed at 6 months of age. WBC counts (A), Hb concentrations (B), and Plt numbers (C) were analyzed using a Hemavet 950FS. The percentages of CD11b^{+} granulocytes/monocytes, B220^{+} B lymphocytes, and CD3^{+} T lymphocytes were analyzed by flow cytometry (D). Representative flow cytometric data (E) and absolute numbers (F) of HSCs and HPCs in the BM of WT, *Ripk3*$^{-/-}$, and *Mlkl*$^{-/-}$ mice are shown.

from third transplantation recipients for CFU assay and competitive transplantation study, we found that, despite the comparable CFU capacity of BM cells among all genotypes (Figure 2G), only *Ripk3*$^{-/-}$ BM cells were able to maintain their competitive hematopoietic reconstitutive capacity (CHRC; as demonstrated by percentage of donor-derived cells in PB), while the CHRC was significantly reduced in both *Mlkl*$^{-/-}$ and WT BM cells (Figure 2H). In addition, significant myeloid-biased hematopoiesis was observed in *Mlkl*$^{-/-}$ and WT BM cells, but not in *Ripk3*$^{-/-}$ BM cells (Figure S3C). This study suggests that Ripk3 signaling impairs HSC function during serial transplantation through an Mlkl-independent mechanism. Consistent with this observation, in a serial competitive transplantation study we also found that the CHRCs of *Ripk3*$^{-/-}$, *Mlkl*$^{-/-}$, and WT BM cells were comparable for the first and second transplantations. However, upon third transplantation, a significant reduction in CHRC was observed in both *Mlkl*$^{-/-}$ and WT BM cells, but not in *Ripk3*$^{-/-}$ BM cells (Figure S3D).

In addition to inducing Mlkl-mediated necroptosis, Ripk3 also directly interacts with mitochondria and induces ROS production (Yang et al., 2018b). We found increased ROS production in both WT and *Mlkl*$^{-/-}$ HSCs but not in *Ripk3*$^{-/-}$ HSCs collected from recipient mice from the third transplantation (Figure 2I), which was correlated with an increase in p-p38 MAPK (Figure 2J). ROS-p38 signaling impairs HSC self-renewal by inducing p16 expression and senescence (Hishiya et al., 2005; Wang et al., 2011). We found significantly increased senescence in both WT and *Mlkl*$^{-/-}$ HSCs but not in *Ripk3*$^{-/-}$ HSCs (Figure 2K), which was associated with an increase in expression of p16, p19, and p15 (Figure 2L). This suggests that Ripk3 impairs HSC functions through induction of ROS production and p38-p16-mediated cellular senescence, both of these independent of Mlkl.

Ripk3 deletion promotes IR-induced leukemia/lymphoma development in mice

Fractionated low-dosage IR (1.75 Gy weekly × 4) induces thymomas (lymphomas in the thymus gland) in WT mice. To study the role of Ripk3-Mlkl signaling in IR-induced leukemia/lymphoma development, we treated WT, *Ripk3*$^{-/-}$, and *Mlkl*$^{-/-}$ mice with 1.75 Gy × 4 IR and monitored for disease development and mouse survival.

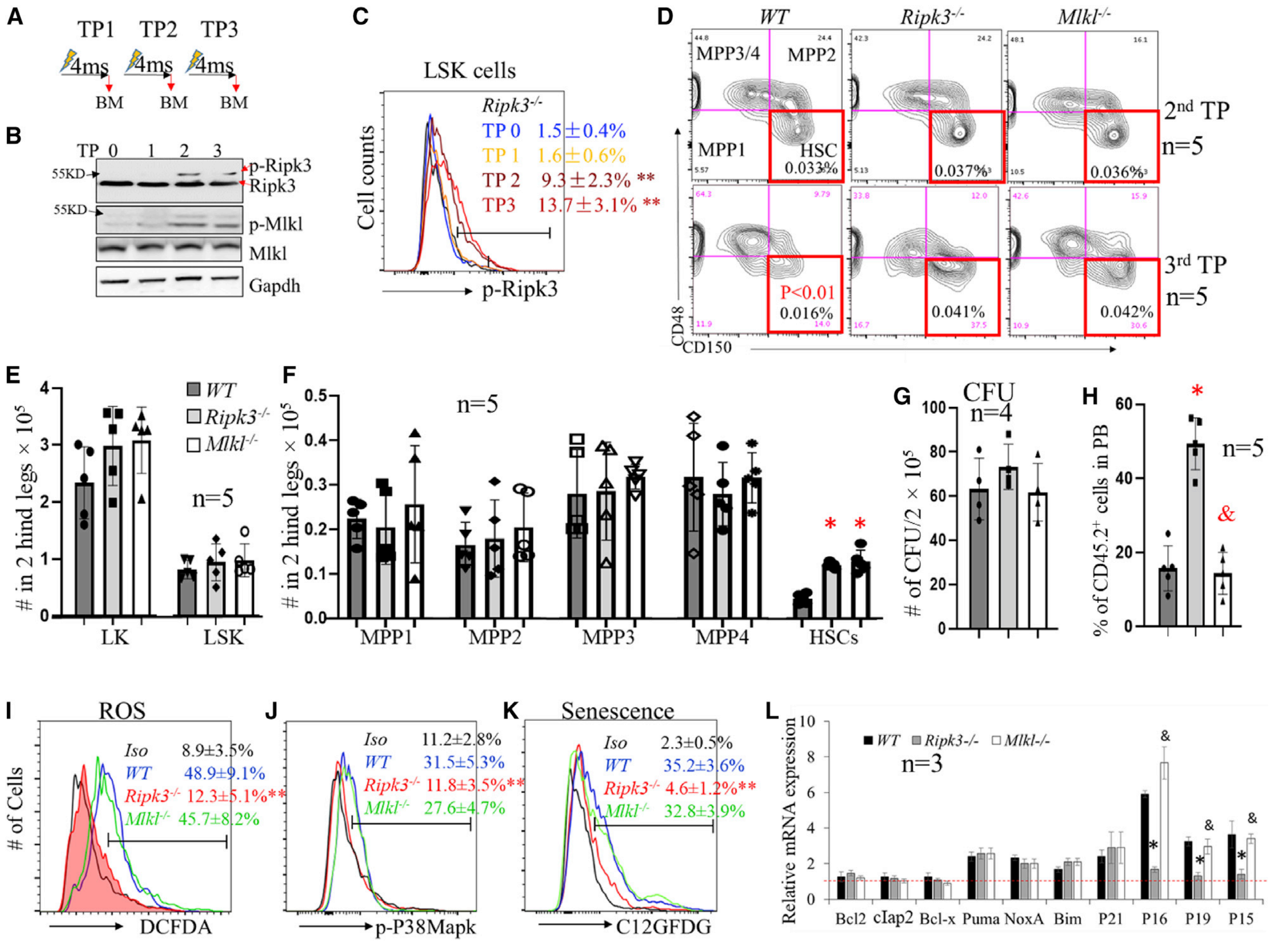

Figure 2. Ripk3 selectively regulates number and function of HSCs during serial transplantation in Mlkl-dependent and -independent manners, respectively

(A) Serial transplantation and analysis schedule. TP0 represents non-transplanted control. TP1, TP2, and TP3 indicate first, second, and third transplantations, respectively. “4ms” represents 4 months post-transplantation.

(B and C) BM MNCs were collected from recipient mice 4 months post-transplantation for each transplantation cycle. Activation of Ripk3-Mlkl signaling in BM MNCs (B) and LSK cells (C) was examined by western blotting and flow cytometric assays, respectively. For flow cytometric analysis, *Ripk3*$^{-/-}$ LSK cells were always used as a negative control to set up the flow cytometer in order to make the data consistent in all of the experiments.

(D) Representative flow cytometric data for HSCs and MPPs among CD45.2^{+} BM MNCs from second and third transplantation recipients of the indicated genotypes of donors.

(E and F) Absolute numbers of LK cells and LSK cells (E) as well as HSCs and MPPs (F) in the BM from third transplantation recipients of the indicated donor genotypes.

(G–L) BM MNCs were collected from third transplantation recipients of the indicated genotypes of donors. The cells were seeded into methylcellulose medium for CFU-C assay. The numbers of colonies were counted after 7 days of culturing (G). Cells were mixed with equal numbers of competitor BM cells for competitive transplantation study. The CHRC of the donor cells was analyzed 4 months after transplantation by examining the percentage of donor-derived cells (CD45.2^{+}) in PB (H). ROS levels (I), p-p38 Mapk levels (J), and senescence (K) in LSK cells were examined by dichlorofluores cin diacetate (DCFDA), p-p38 antibody, and 5-Dodecanoylaminofluorescein Di-β-D-Galactopyranoside (C12GFDG) staining, respectively, followed by flow cytometric analysis. Data in (C), (I), (J), and (K) show one of the three biological triplicate experiments. “Iso” stands for isoform control. The expression of the indicated genes in LSK cells of the indicated genotypes was examined by qRT-PCR assay and normalized to the levels of the same gene in LSK cells isolated from non-transplanted *WT* mice (L). $^{*}p < 0.05$ and $^{**}p < 0.01$, compared with WT group or TP0 group. $^{\&}p < 0.05$, compared with *Ripk3*$^{-/-}$ group.

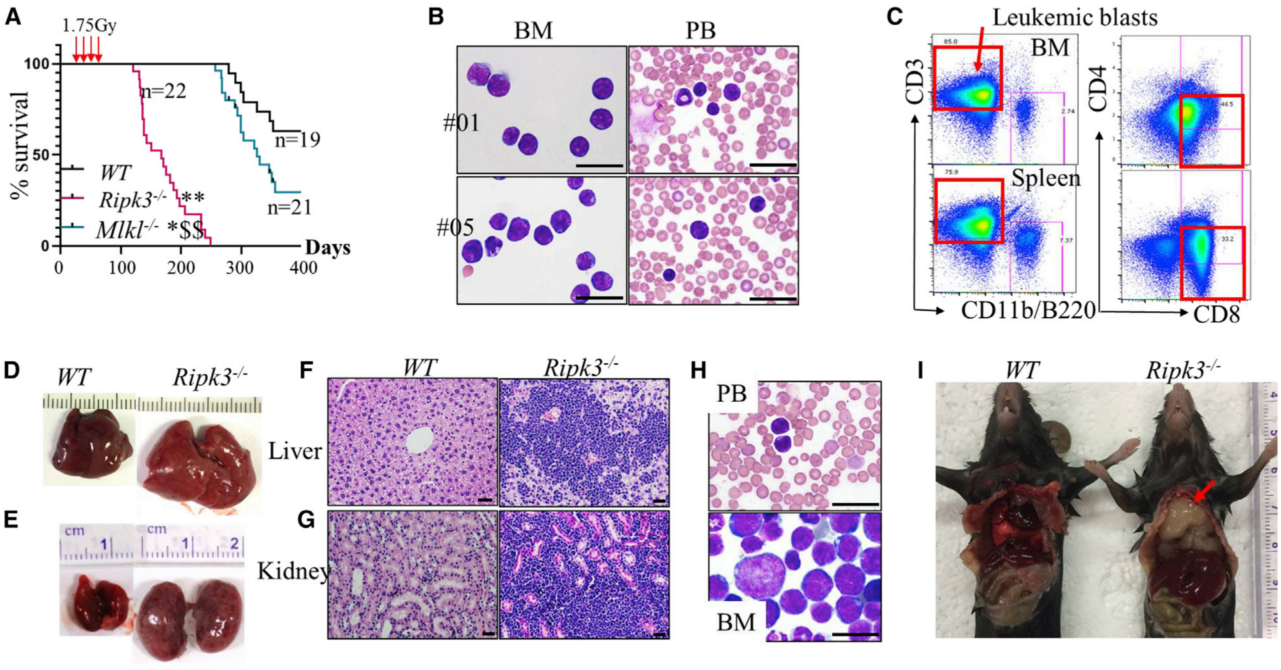

Figure 3. Inactivation of Ripk3 signaling promotes IR-induced leukemia in mice

WT, Ripk3$^{-/-}$, and *Mlkl*$^{-/-}$ mice were X-irradiated at 1.75 Gy weekly × 4. The mice were monitored for leukemia development.

(A–C) Survival curves for the mice were plotted by Kaplan-Meier graphing (A). Leukemia in the mice was diagnosed by examination for leukemic blasts in BM and PB by morphology (B) and flow cytometry (C).

(D–G) Leukemia was further verified by liver and kidney infiltration of leukemic blasts.

(H) Mice transplanted with 1 × 10^6 leukemic cells from *Ripk3*$^{-/-}$ mice developed the same types of leukemia as donor mice as demonstrated by morphologic analysis of leukemic blasts in the PB and BM.

(I) Thymoma (indicated by arrow) developed in *Ripk3*$^{-/-}$ mice but not in WT mice by 200–250 days post-IR. Scale bars in (B), (F), (G), and (H) represent 50 μm.

Most (77%, 17/22) of the *Ripk3*$^{-/-}$ mice developed acute T lymphoblastic leukemia (T-ALL) and died within 200 days, while the remaining *Ripk3*$^{-/-}$ mice developed a mixture of T-ALL/thymoma and died within 250 days (Figure 3A). T-ALL was diagnosed as >30% CD8$^+$CD3$^{+/-}$ T lymphoblasts in BM and PB (Figures 3B and 3C), as well as liver and kidney infiltration of lymphoblasts (Figures 3D–3G). In addition, T-ALL cells collected from BM or spleens of the mice were able to generate the same type of T-ALL in recipient mice upon transplantation (Figure 3H). However, 36.8% (7/19) of WT mice and 76.2% (16/21) of *Mlkl*$^{-/-}$ mice died of thymoma within 360 days (Figures 3A and 3I). *Mlkl* deletion only slightly promoted the development of IR-induced thymoma. These data suggest that Ripk3 signaling represses T-ALL development and delays the development of thymomas primarily through an Mlkl-independent mechanism. Because the lymphoblasts collected from both T-ALL and thymomic mice were phenotypically the same (CD8$^+$CD3$^{+/-}$), we speculate that the T-ALL and thymoma might be the same type of disease.

Ripk3-Mlkl signaling inactivation prevents low-dose IR-induced HSC exhaustion

To study the mechanism by which Ripk3 signaling represses T-ALL development, we compared IR-induced DNA damage repair in HSCs isolated from WT, *Ripk3*$^{-/-}$, and *Mlkl*$^{-/-}$ mice. As expected, a significantly increased percentage of γ-H2A.X$^+$ cells and greater numbers of γ-H2A.X$^+$ foci/cell were detected among all three genotypes of LSKs by 2 h after IR. Both the percentages of γ-H2A.X$^+$ cells and the numbers of γ-H2A.X$^+$ foci/cell were reduced to levels slightly higher than basal at 48 h post-IR. No significant differences were observed among all three genotypes of mice, suggesting that inactivation of Ripk3-Mlkl signaling did not affect DNA damage repair in HSCs (Figures 4A and 4B).

We then compared the numbers of HPCs and HSCs among WT, *Ripk3*$^{-/-}$, and *Mlkl*$^{-/-}$ mice 1 month after 1.75 Gy × 4 IR. Although LK-MPs, LSKs, and MPPs were comparable among all three genotypes of mice, a significant reduction in HSCs was observed in WT mice, suggesting that Ripk3 signaling and consequent Mlkl-mediated

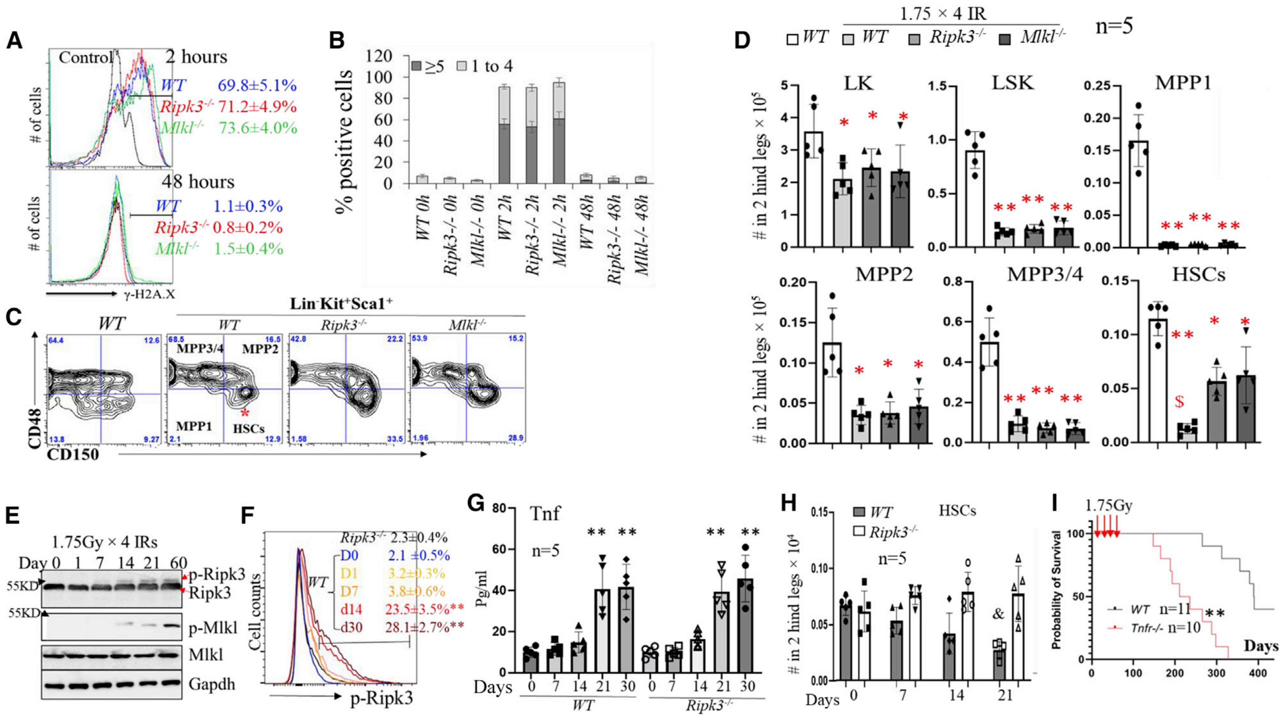

Figure 4. Ripk3 signaling promotes low-dose IR-induced HSC elimination in an Mlkl-dependent manner

(A and B) WT, *Ripk3*$^{-/-}$, and *Mlkl*$^{-/-}$ mice were irradiated with 6 Gy X-ray. BM MNCs were collected 2 and 48 h after IR. DNA damage was examined in LSK cells by γ-H2A.X staining followed by flow cytometry (A) and microscopic analysis (B).

(C and D) WT, *Ripk3*$^{-/-}$, and *Mlkl*$^{-/-}$ mice were irradiated with 1.75 Gy X-ray weekly × 4. BM MNCs were collected from the mice 1 month following the last IR. HSCs and HPCs were analyzed by flow cytometry gating on LSK and LK populations. Representative flow cytometric data (C) and absolute numbers of HSCs and HPCs (D) in the BM of the indicated genotypes of mice are presented. Five mice were studied in each group. $^{\$}p < 0.05$ compared with Ripk3$^{-/-}$ or Mlkjl$^{-/-}$ groups.

(E–H) WT and *Ripk3*$^{-/-}$ mice were irradiated with X-rays, 1.75 Gy, every week for a maximum of 4 weeks. BM and PB were collected from the mice at the indicated times after the first IR. Ripk3-Mlkl signaling in BM MNC and LSK populations from WT mice was examined at the indicated times after the first IR by western blotting (E) and flow cytometry (F), respectively. Data in (F) show one of the three biological triplicate experiments, and LSK cells from *Ripk3*$^{-/-}$ mice were always studied in parallel as controls. Levels of Tnf-α in PB were examined by ELISA (G). HSC numbers/two hindlegs were examined by flow cytometric analysis (H).

(I) WT and *Tnfr*$^{-/-}$ mice were irradiated with 1.75 Gy X-ray weekly × 4. Mice were monitored for leukemia development. Survival curves for the mice were plotted by Kaplan-Meier graphing. $^{*}p < 0.05$ and $^{**}p < 0.01$, compared with non-irradiated WT mice or D0 group. $^{\&}p < 0.05$, compared with irradiated *Ripk3*$^{-/-}$ or *Mlkl*$^{-/-}$ mice. In (G), $^{**}p < 0.01$ compared with days 0, 1, and 7.

necroptosis promotes the loss of HSCs induced by low-dose IR (Figures 4C, 4D, and S4A–S4C). In the chimeric transplantation model (we used a 1:1 ratio of WT and Ripk3$^{-/-}$ BM cells here), 1.75 Gy × 4 IR treatment could reduce only the number of WT HSCs and not *Ripk3*$^{-/-}$ HSCs, suggesting a cell-autonomous role for Ripk3 signaling in stress-induced HSC depletion (Figure S4D). Detailed analysis demonstrated that in WT mice, low-dose IR induced the activation of Ripk3 signaling as early as 2 weeks after the first IR in BM MNCs as shown by increased levels of p-Ripk3 and p-Mlkl (Figure 4E), as well as in LSK-HSPCs (Figure 4F) and HSCs (Figure S4E) as shown by increased levels of p-Ripk3. The activation of Ripk3 signaling was correlated with increased levels of Tnf-α in PB (Figure 4G), which was followed by the reduction in HSCs as early as the third week after the first exposure to IR (Figure 4H). Interestingly, Tnf-α levels were also elevated in the PB of IR-treated *Ripk3*$^{-/-}$ mice (Figure 4G); however, p-Mlkl levels were not increased in BM MNCs of IR-treated *Ripk3*$^{-/-}$ mice (data not shown). This suggests that IR induces Ripk3-independent Tnf-α production but a Ripk3-dependent activation of Mlkl signaling. In addition, 1.75 Gy × 4 IR failed to induce the activation of Ripk3 in HSCs in *Tnfr1*$^{-/-}$ mice (Figure S5A). As was the case with *Ripk3*$^{-/-}$ mice (Figure 4H), significantly more HSCs were retained in *Tnfr1*$^{-/-}$ mice compared with WT mice following 1.75 Gy × 4 IR (Figure S5A). Finally, IR-induced leukemia development was significantly accelerated in

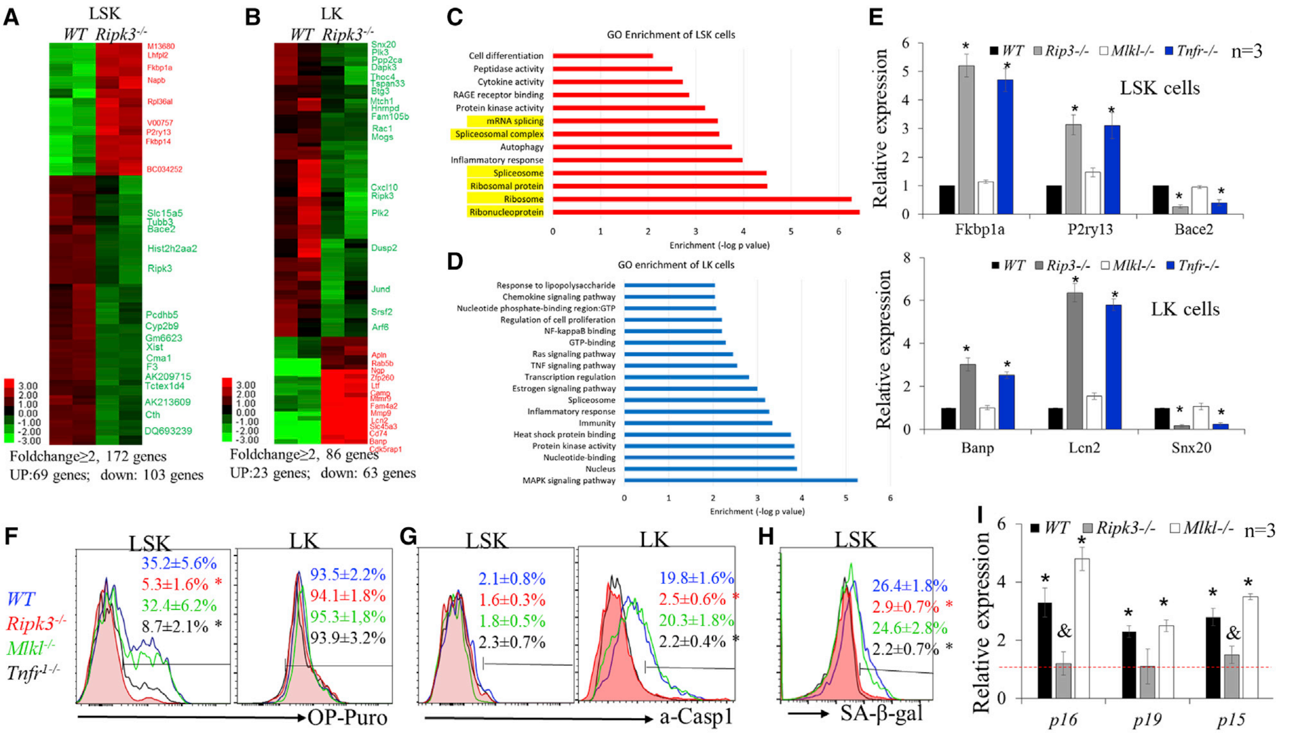

Figure 5. Ripk3 signaling induces protein synthesis and cellular senescence in HSCs in an Mlkl-independent fashion

(A–D) WT and *Ripk3*$^{-/-}$ mice were irradiated with X-rays, 1.75 Gy weekly × 4. LK and LSK cells were collected from mouse BM 1 month after the final IR. Gene expression was examined by RNA-seq. Genes up- or downregulated in LK and LSK cells of *Ripk3*$^{-/-}$ mice compared with WT mice are presented in the heatmaps (A and B). Signaling pathways specifically altered in the LSK and LK populations of *Ripk3*$^{-/-}$ mice compared with WT mice were analyzed by GO enrichment analysis (C and D).

(E–I) WT, *Ripk3*$^{-/-}$, *Mlkl*$^{-/-}$, and *Tnfr*$^{-/-}$ mice were irradiated with X-rays, 1.75 Gy weekly × 4. LK and LSK cells were collected from mouse BM 1 month after the last IR. The expression of selected genes from RNA-seq data was verified in LSK and LK cells by qRT-PCR (E). Protein synthesis (F), inflammasome activity (G), and senescence (H and I) were examined by OP-puro, a-Casp1, and C12GFDG staining, as well as p16, p19, and p15 expression (I). Data in (F), (G), and (H) show one of the three biological triplicate experiments. *$p < 0.01$, compared with non-irradiated controls. $^{\&}p < 0.05$, compared with WT and *Mlkl*$^{-/-}$ mice.

Tnfr1$^{-/-}$ mice (Figure 4I). Taken together, these studies suggested that Ripk3 signaling in HSCs is primarily stimulated by the inflammatory cytokine Tnf-α during fractionated low-dose IR exposures. Nevertheless, enhanced T-ALL development was observed only in *Ripk3*$^{-/-}$ mice and not in *Mlkl*$^{-/-}$ mice, suggesting that the tumor-repressive activity of Ripk3 is primarily independent of *Mlkl*-mediated HSC elimination.

Low-dosage IR promotes Tnf-α-induced protein synthesis in HSCs in a Ripk3-dependent but Mlkl-independent manner

To study the Mlkl-independent tumor-repressive mechanism of Ripk3 signaling, we compared gene expression profiles among LK and LSK cells isolated from WT and *Ripk3*$^{-/-}$ mice 1 month after the fourth in a series of IRs using RNA-sequencing (RNA-seq) assay. We found that 86 genes showed ≥2-fold change (upregulation of 23 genes and downregulation of 63 genes) in *Ripk3*$^{-/-}$ LK cells compared with WT LK cells, while 172 genes showed ≥2-fold change (upregulation of 69 genes and downregulation of 103 genes) in *Ripk3*$^{-/-}$ LSK cells compared with WT LSK cells (Figures 5A and 5B). Interestingly, most of the genes altered in LSK cells did not overlap with genes altered in LK cells. Gene Ontology (GO) enrichment analysis demonstrated that the altered pathways in LK cells were primarily involved in TNF/LPS-stimulated NF-κB and MAPK signaling, while the pathways altered in LSK cells were mainly involved in ribosomal and mRNA splice signaling (Figures 5C and 5D). qRT-PCR assay demonstrated that gene expression changes in LSKs and LKs isolated from *Mlkl*$^{-/-}$ mice were similar to those in the corresponding type of cells isolated from WT, while the gene changes in LSKs and LKs from *Tnfr*$^{-/-}$ mice were similar to those in the corresponding type of cells from *Ripk3*$^{-/-}$ mice (Figure 5C). Consistent with this observation, we found an increased rate of protein synthesis in LSK-HSPCs isolated from IR WT and *Mlkl*$^{-/-}$ mice but not in LSK-HSPCs

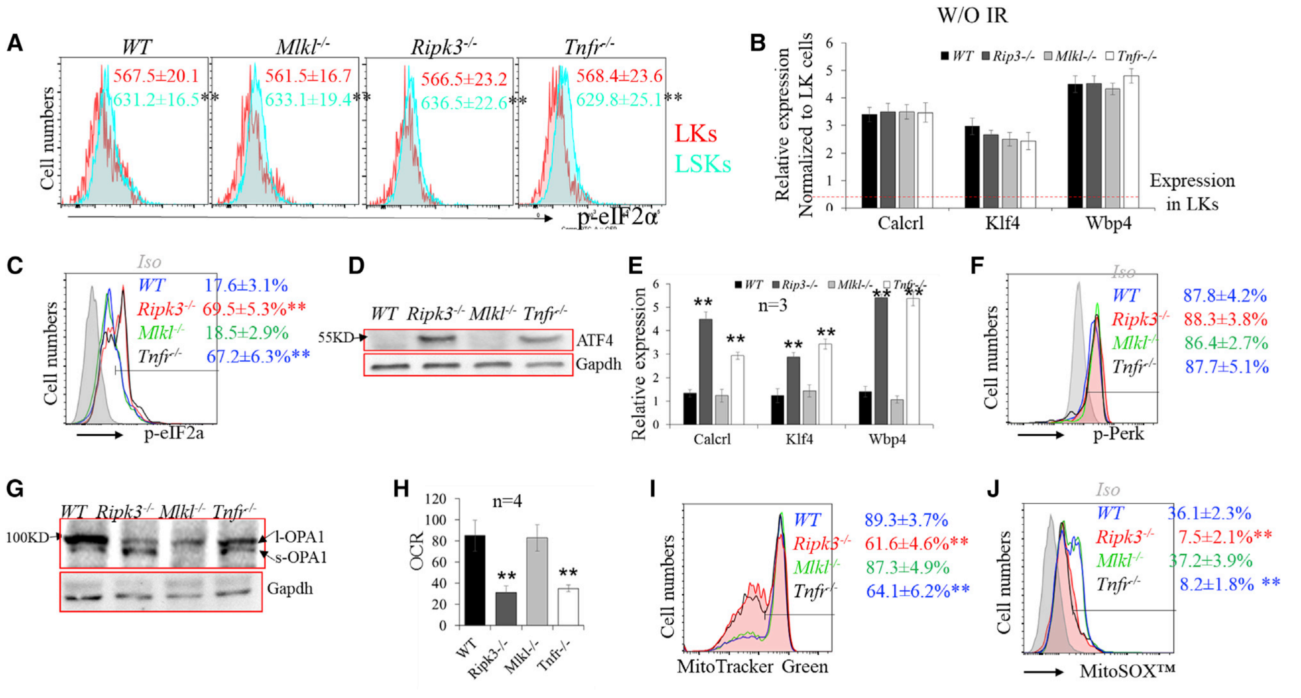

Figure 6. Ripk3 signaling induces protein synthesis and cellular senescence in HSCs by stimulating PDC-mediated OXPHOS and attenuating mitochondrial ISR

(A and B) The basal activity of eIF2a-Atf4 signaling in WT, *Ripk3*$^{-/-}$, *Mlkl*$^{-/-}$, and *Tnfr*$^{-/-}$ HSCs was examined by flow cytometry for p-eIF2a levels (mean fluorescence intensity, MFI) (A) and qRT-PCR for the expression of Atf4 target genes (B).

(C–J) WT, *Ripk3*$^{-/-}$, *Mlkl*$^{-/-}$, and *Tnfr*$^{-/-}$ mice were irradiated with X-rays, 1.75 Gy weekly × 4. LSK cells were collected from mouse BM 1 month after the final IR. The activity of eIF2a-Atf4 signaling was examined by flow cytometry for p-eIF2a levels (C), western blotting for ATF4 expression (D), and qRT-PCR for the expression of Atf4 target genes (E). The activity of Perk-ER stress signaling was examined by p-Perk levels (F). The activity of OMA1 was examined by western blotting for OPA cleavage (G); OXPHOS was examined by OCR (H); and mitochondrial mass and mtROS were examined by MitoTracker green staining (I) and MitoSOX red staining (J). Data in (A), (C), (F), (I), and (J) show one of the three biological triplicate experiments. $^{**}p < 0.01$ compared with LK in (A) or WT and *Mlkl*$^{-/-}$ mice.

isolated from irradiated *Tnfr*$^{-/-}$ and *Ripk3*$^{-/-}$ mice as determined by OP-puro label assay; however, the protein synthesis rates in LK-MPs from all four genotypes of mice were comparable (Figure 5F). In addition, we found increased inflammasome activity in LK-MPs isolated from irradiated WT and *Mlkl*$^{-/-}$ mice compared with LK-MPs isolated from irradiated *Tnfr*$^{-/-}$ and *Ripk3*$^{-/-}$ mice, as demonstrated by increased activated Caspase 1 (a-Casp1) (Figure 5G); however, inflammasome activity in LSK-HSPCs from all four genotypes of mice was not altered (Figure 5G). This suggests that HSPCs and MPs have distinct responses to Tnf-α-stimulated Ripk3 signaling resulting from low-dosage IR. Maintaining a low level of protein synthesis in HSCs is critical for preserving their self-renewal and hematopoietic reconstitutive capacities (Hidalgo San Jose et al., 2020; Signer et al., 2014). As a consequence, we detected increased senescence in both WT and *Mlkl*$^{-/-}$ HSCs compared with *Ripk3*$^{-/-}$ and *Tnfr*$^{-/-}$ HSCs as determined by both β-gal staining (Figure 5H) and p16/p19 expression (Figure 5I). Thus we speculate that Ripk3 mediates a Tnf-α-stimulated tumor-repressing activity through induction of protein synthesis and cellular senescence in HSCs.

Low-dose IR impairs mitochondrial-integrated stress response (ISR) in HSCs in a Ripk3-dependent but Mlkl-independent manner

The rate of protein synthesis in HSCs is restricted by eIF2a-Atf4 pathway-mediated ISR. To study whether the increased protein synthesis observed in WT and *Mlkl*$^{-/-}$ HSCs is due to the attenuation of the eIF2-Atf4 ISR pathway, we compared the activity of the eIF2α-Atf4 pathway among LSK and LK cells isolated from WT, *Ripk3*$^{-/-}$, *Mlkl*$^{-/-}$,and *Tnfr*$^{-/-}$ mice before and after 4× IR. We found that, prior to IR, the activity of the eIF2α-Atf4 pathway is higher in LSK cells compared with LK cells isolated from all four genotypes of mice, as determined by increased levels of p-eIF2α and elevated expression of Atf4 target genes (Figures 6A and 6B). No difference was observed among the four genotypes of mice. However, after

IR, such activity was induced in LSK cells isolated from *Ripk3*$^{-/-}$ and *Tnfr*$^{-/-}$ mice, but was not altered in LSK cells isolated from WT and *Mlkl*$^{-/-}$ mice (Figures 6C–6E). No change was observed in LK cells from any of the genotypes of mice (Figure S6).

Four eIF2α kinases, GCN2, PERK, PKR, and HRI, can phosphorylate eIF2α and activate eIF2α-Atf4-ISR signaling. GCN2 is stimulated by amino acid depletion and PKR is activated by double-stranded RNA during viral infection. However, basal levels of eIF2α phosphorylation are primarily controlled by either endoplasmic reticulum (ER) stress-related PERK or mitochondrial stress-related HRI signaling (Fessler et al., 2020; van Galen et al., 2018). We found that there is no difference in PERK signaling among our four genotypes of LSK cells, as demonstrated by comparable levels of p-Perk (Figure 6F) and the expression of Grp78, an indicator of ER stress (data not shown). Thus we investigated whether the attenuated eIF2α-Atf4 ISR signaling in WT and *Mlkl*$^{-/-}$ HSCs is due to the failure of the OMA1–DELE1–HRI-mediated mitochondrial stress response (Fessler et al., 2020). In this pathway, DELE1 is localized to the inner membrane of mitochondria and is cleaved by active OMA1. The cleaved DELE1 is then released into the cytoplasm, where it activates HRI (Fessler et al., 2020). Due to the lack of antibodies to directly detect DELE1 and active HRI, we examined OMA1 activity by detecting the cleavage of the dynamin-like GTPase OPA1, a well-known OMA1 substrate. OMA1 is constitutively active in HSCs (van Galen et al., 2018). We found a significant reduction of OMA1 activity in WT and *Mlkl*$^{-/-}$ HSCs compared with *Ripk3*$^{-/-}$ and *Tnfr*$^{-/-}$ HSCs as determined by a reduction in the s-OPA1/l-OPA1 ratio (Figure 6G).

Pyruvate dehydrogenase complex (Pdc) is the rate-limiting enzyme that converts pyruvate to acetyl-CoA, linking glycolysis to aerobic respiration (Hitosugi et al., 2011; Kaplon et al., 2013). Tnf-α induces Ripk3-dependent aerobic mitochondrial metabolism, oxidative phosphorylation (OXPHOS), and ROS production in many types of cells by stimulating the Ripk3-mediated phosphorylation of Pdc-E3, a key subunit of Pdc (Qiu et al., 2018; Yang et al., 2018a, b). Pdc has been described as a key mediator of oncogene-induced senescence, a vital pathophysiological mechanism that protects against cancer (Kaplon et al., 2013). We found increased OXPHOS in WT and *Mlkl*$^{-/-}$ HSCs compared with *Ripk3*$^{-/-}$ and *Tnfr*$^{-/-}$ HSCs, as determined by increased oxygen consumption rate (OCR) (Figure 6H), which was associated with increased mitochondrial mass and mitochondrial ROS (mtROS), as demonstrated by MitoTracker green and MitoSOX red staining (Figures 6I and 6J).

To study whether the impaired eIF2α-Atf4 ISR signaling in WT and *Mlkl*$^{-/-}$ HSCs was due to increased Pdh-OXPHOS-mtROS, we treated irradiated mice with either the Pdh inhibitor CPI-613 or the ROS scavenger N-acetyl-L-cysteine (NAC). We found that either CPI-613 or NAC treatment could inhibit OXPHOS and mtROS and restore OPA1 cleavage and eIF2α-ATF4 signaling in both WT (Figures 7A–7E) and *Mlkl*$^{-/-}$ HSCs (Figures 7A and 7B). As a consequence, protein synthesis and senescence were repressed in both WT (Figures 7F and 7G) and *Mlkl*$^{-/-}$ HSCs (Figures S7C and S7D). Finally, CPI-613 treatment also promotes the development of T-ALL/lymphomas in WT mice (Figure 7H). In the CPI-613 treatment group, 1/12 mice died of T-ALL and 4/12 mice died of a mixture of T-ALL/thymoma within 240 days, while the remaining mice died of thymoma within 450 days. However, in the vehicle-treated group, only 5/11 mice died of thymoma within 500 days.

DISCUSSION

Ripk3-mediated necroptosis is a hallmark feature of cellular clearance of infection and plays a key role in immunity and inflammation. Abnormal activation of Ripk3 signaling is involved in many aging-related diseases, such as atherosclerosis, stroke, and leukemia. In this study, we describe a novel and HSC-specific function for Ripk3 signaling in stress hematopoiesis. We found that Ripk3 signaling was not activated in HSCs during normal homeostasis but was induced during stress hematopoiesis, such as serial transplantation and fractionated low-dose exposure to IR. Such stresses induced a delayed chronic activation of Ripk3-Mlkl signaling, which selectively regulated both the quantity of HSCs by stimulating Mlkl-dependent necroptosis and the quality of HSCs by triggering Mlkl-independent senescence.

We found that the delayed chronic activation of Ripk3-Mlkl signaling in HSCs during serial transplantation or fractionated low-dose IR exposure was largely induced by Tnf-α. Early studies suggested that Tnf-α treatment represses IR-induced leukemia by inhibiting pre-LSCs (Boniver et al., 1989; Humblet et al., 1996). However, the underlying molecular mechanism to explain this is still unknown. A recent study suggested that Tnf-α selectively regulates HSC numbers during inflammation by means of Ripk3-Mlkl-mediated necroptosis (Yamashita and Passegue, 2019). We found that, during both serial transplantation and IR exposure, in addition to inducing Mlkl-necroptosis, thus reducing the number of HSCs, Tnf-α attenuates HSC functioning and represses IR-induced leukemia development primarily by stimulating Ripk3 signaling-mediated Mlkl-independent senescence. Thus we believe that during the early pre-leukemic stage, induced activation of Ripk3 signaling might be a useful strategy to repress subsequent leukemic transformation.

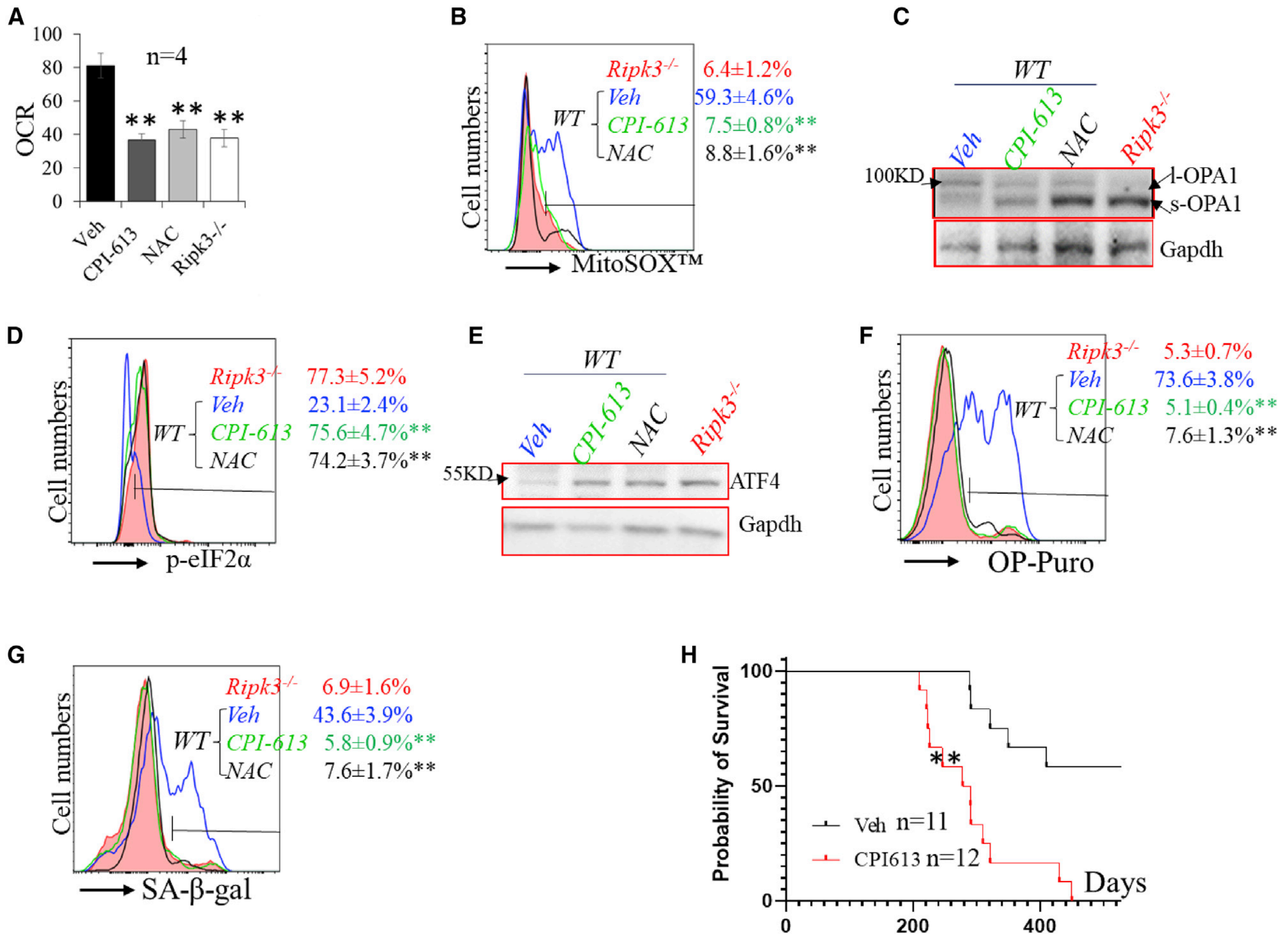

Figure 7. Inhibition of PDH or ROS restores eIF2α-Atf4 ISR signaling in HSCs of irradiated WT mice

(A–G) WT mice were irradiated with X-rays, 1.75 Gy weekly × 4, and treated with vehicle (Veh), CPI-613, or NAC. LSK cells were collected from mouse BM 1 month after the last IR. LSKs isolated from irradiated *Ripk3*$^{-/-}$ mice were studied as controls. OCR (A), mtROS (B), OPA cleavage (C), p-eIF2a (D), ATF4 expression (E), rate of protein synthesis (F), and senescence (G) were examined.

(H) BM MNCs were collected 1 week after the last IR and transplanted into lethally irradiated mice. Recipient mice were monitored for leukemia development. Survival curves for the mice were plotted by Kaplan-Meier graphing. Data in (B), (D), (F), and (G) show one of the three biological triplicate experiments. **$p < 0.01$, compared with respective vehicle controls.

Sublethal IR exposure induces p53-dependent cell-cycle arrest/senescence in HSCs (Domen et al., 1998; Wang et al., 2006). Sustained impairment of HSCs is the cause of LT-RD, which is associated with increased biomarkers of cellular senescence and a prolonged elevation of p21$^{Cip1/Waf1}$, p19^{Arf}, and p16^{Ink4a} expression. However, deletion of both *p19*^{Arf} and *p16*^{Ink4a} genes failed to prevent senescence in HSCs, suggesting that p21$^{Cip1/Waf1}$ may play an important role in the senescence of HSCs following sublethal doses of IR (Shao et al., 2014). It was reported that Ripk3 signaling stimulates mtROS production by enhancing aerobic metabolism and respiration and attenuating mitochondrial ISR (van Galen et al., 2018; Yang et al., 2018b). We found that during fractionated low-dose IR exposure, Tnf-α-Ripk3 signaling stimulates PDC-mediated aerobic respiration and mtROS production and attenuates mitochondrial ISR, leading to increased protein synthesis and cellular senescence in pre-LSCs. Whether such Ripk3 signaling-mediated senescence in HSCs is also p53 dependent remains to be determined.

HSCs and HPCs do not respond to IR treatment in the same way (Milyavsky et al., 2010). A sublethal dose of IR (6.0–6.5 Gy) selectively kills proliferative HPCs by activating p53/Puma-mediated apoptosis but causes cell-cycle arrest and senescence in HSCs (Domen et al., 1998; Wang et al., 2006). IR induces the development of leukemia by inducing DNA damage in HSCs. Double-strand breakage is the most common type of DNA damage induced by IR. Unlike proliferative HPCs, which use homologous recombination, an error-proof mechanism to repair their

damaged DNA, HSCs primarily use non-homologous end joining, an error-prone DNA repair mechanism, to repair their damaged DNA (Mohrin et al., 2010). Thus significant numbers of mutations will develop in HSCs after IR exposure. Fortunately, most of the mutant HSCs remain in a senescent state, which might represent a condition associated with pre-malignancy (Campisi and d'Adda di Fagagna, 2007). Only a few mutant HSCs that undergo intrinsic epigenetic and/or metabolic reprogramming and escape from cell-cycle arrest will acquire leukemia-initiating potential (Aird et al., 2015; Kang et al., 2017; Milanovic et al., 2018; Mosteiro et al., 2016; Ritschka et al., 2017). Thus p16-mediated senescence is one of the essential barriers to protect against IR- or oncogene-induced leukemogenesis (Palacio et al., 2017). This explains why deletion or hypermethylation of the *p16*^{INK4a} locus is commonly detected in patients with B-ALL or T-ALL (Malumbres et al., 1999; Ohnishi et al., 1995; Sulong et al., 2009). However, LSCs in AML are more like HPCs than HSCs. We found that IR-induced Tnf-α-Ripk3 signaling plays distinctly different roles in HSCs and HPCs. In HSCs, Tnf-α-Ripk3 signaling stimulates mitochondrial OXPHOS and attenuates ISR, which leads to increased protein synthesis and cellular senescence. However, in HPCs, Tnf-α-Ripk3 signaling stimulates the activation of inflammasomes, which might explain the critical role of Tnf-α-Ripk3-inflammasome signaling in the pathogenesis of *RUNX1-ETO*- or FLT3-ITD-induced AML in models (Hockendorf et al., 2016; Xin et al., 2016b). Yang et al. reported that Tnf-α-Ripk3 signaling increases mitochondrial OXPHOS by phosphorylating Pdc-E3 and activating Pdh in multiple human cell lines as well as murine embryonic fibroblasts (Yang et al., 2018b). Consistently, we found that IR-induced Tnf-α-Ripk3 signaling also stimulates Pdh activation and mitochondrial OXPHOS in HSCs. Yang's study demonstrated that Tnf-α-Ripk3 signaling-stimulated mitochondrial OXPHOS in L929 cells is dependent upon Mlkl (Yang et al., 2018b). However we found that Mlkl is not required for the Tnf-α-Ripk3 signaling-stimulated mitochondrial OXPHOS in HSCs. We speculate that such a discrepancy might be due to differences in the way in which signaling is activated and/or the nature of the specific cell type studied.

EXPERIMENTAL PROCEDURES

Mice

Ripk3$^{-/-}$ mice, *Mlkl*$^{-/-}$ mice, and *Tnfr1*$^{-/-}$ mice, described previously, were maintained in a C57BL/6J background (Wang et al., 2016; Xin et al., 2016b). WT C57BL/6J control mice and Ptprc recipient mice were purchased from The Jackson Laboratory (Bar Harbor, ME). All mice were maintained according to the standards in the National Institutes of Health *Guidelines for the Care and Use of Animals* in the AAALAC-certified pathogen-free animal facility at Loyola University Medical Center. All mice were housed under a 12-h light/dark cycle in microisolator cages contained within a laminar flow system. All procedures were conducted in accordance with the National Institutes of Health guidelines for the care and use of laboratory animals for research purposes and were approved in advance by the Loyola University Chicago IACUC (2017-038). Genotypes of the mice were determined by PCR assay. The PCR primer sequences for genotyping can be found in Table S1.

Mouse irradiation and treatments

Mice were exposed to various doses of IR in an RS 2000 biological research X-ray irradiator (Rad Source Technologies) at a rate of 1.17 Gy/min. Mice were irradiated in a specifically designed mouse cage, four mice per cage. After radiation, all mice were housed in a germ-free facility and provided with drinking water containing 0.16 mg/mL Baytril for 2 weeks to prevent bacterial infection.

To study IR-induced leukemia development, mice were treated with 1.75 Gy × 4 IR and monitored for leukemia development. Survival curves of mice were calculated by Kaplan-Meier estimation. The PDC inhibitor CPI-613 was purchased from InvivoChem (V0853), dissolved in 1% DMSO/30% polyethylene glycol/1% Tween 80, and administered intraperitoneally (i.p.), 20 mg/kg, every other day for 3 weeks. NAC was purchased from Sigma-Aldrich and administered at 200 mg/kg daily for 3 consecutive weeks. The p38 MAPK inhibitor SB 203580 was purchased from AdooQ, dissolved in PEG 400/0.5% Tween 80/5% propylene glycol, and administered at a dosage of 5 mg/kg/daily i.p.

Mouse hematopoietic phenotype analysis

Mice were sacrificed at the indicated time points to collect PB, spleens, thymuses, and BM. PB was analyzed for WBC counts, Plt, RBC counts, and Hb concentration using a Hemavet 950FS (Drew Scientific). After lysis of RBCs, MNCs from PB, spleens, thymuses, and BM were counted and further stained with cell surface markers for phenotypic analysis using flow cytometry as described previously (Tang et al., 2008; Xiao et al., 2011). All of the fluorescent antibodies used in flow cytometric analyses were purchased from either eBioscience or Biolegend. For HSC and HPC analysis, 1×10^6 BM MNCs were incubated with BV421-conjugated antibodies against lineage$^+$ cells (including CD3ε, B220, Gr-1, CD11b, and Ter-119), anti-Sca-1-PE, anti-c-kit-APC, anti-CD48-APCcy7, anti-CD150-PEcy7, anti-CD135-Alex 710, anti-CD127-Alex 710, anti-CD34-Alex 710, and anti-CD16/32-FITC antibodies. For each sample, a minimum of 300,000 cells was collected on a Fortessa flow cytometer (Fortessa, Becton Dickinson) and the data were analyzed using FlowJo software (FlowJo) after gating on viable single cells.

Isolation of HSCs, MPPs, LSKs, and LK-MPs

BM-MNCs were collected from mice. Lin$^-$ cells were enriched using the MojoSort mouse hematopoietic progenitor cell isolation kit (Biolegend cat. no. 480004) following the protocol provided by the vender. Lin$^-$ cells were then stained with a BV421-conjugated cocktail of anti-lineage antibodies (mixture of anti-Gr1-BV421, anti-CD11b-BV421, anti-CD3-BV421, anti-B220-BV421, and anti-Ter119-BV421), anti-Sca-1-PE, anti-c-kit-APC, anti-CD150-PEcy7,

anti-CD 48-APCcy7, and anti-CD135-Alex 710 after pre-incubation with anti-CD16/32 to block the Fcγ receptors. After being washed, the cells were resuspended in PBS and sorted using a BD FACSAria III sorter (Becton Dickinson, San Jose, CA).

Real-time RT-PCR analysis

Total RNA was isolated from HSCs and MPP, LSK, and LK cells using TRIzol reagent (Invitrogen, Carlsbad, CA) following the manufacturer's protocol. cDNA was generated from RNA using SuperScript III reverse transcriptase (Life Technologies). Levels of mRNA of the genes of interest were examined by qRT-PCR using the TaqMan assay (Thermo Fisher Scientific) following the instructions provided by the vendor. Gapdh was used as a control. The primers for qRT-PCR used in this study are listed in Table S2. The threshold cycle values (CT) for each reaction were determined and averaged using TaqMan SDS analysis software (Applied Biosystems). The changes in target gene expression were calculated by the comparative CT method (fold change = 2[−ΔΔCT]), as described previously. Each sample was a mixture of LSK HSCs from three mice of the same phenotype. Triplicate RT-PCRs were performed.

Statistical analyses

Data are expressed as the mean ± SD. Two-way ANOVA (multiple groups) and Student's t test (two groups) were performed to determine the statistical significance of differences among and between experimental groups. A $p < 0.05$ was considered significant. All analyses were done using GraphPad Prism from GraphPad Software (San Diego, CA).

Data and code availability

The RNA-seq dataset was deposited into the Gene Expression Omnibus archive (accession no. GSE70899 and GEO no. GSE139143).

SUPPLEMENTAL INFORMATION

Supplemental information can be found online at https://doi.org/10.1016/j.stemcr.2022.04.009.

AUTHOR CONTRIBUTIONS

L.Z., H.L., H-M.N., S.L., H.X., M.S., Jun Zhang, W.W., and P.B. performed the research experiments and analyzed the data. P.B., M.D., W.S., W.D., S.H., and Jiwang Zhang analyzed the data and wrote the paper. Jiwang Zhang designed and performed the research, analyzed the data, and wrote the paper. P.B. revised and edited the paper.

CONFLICTS OF INTEREST

The authors declare no competing interests.

ACKNOWLEDGMENTS

The authors thank the staff of the Department of Comparative Medicine at Loyola University Medical Center for their excellent animal care services, as well as Ms. Patricia Simms for flow cytometric sorting of HSCs and progenitors. This work was supported by NIH grants NHLBI R01 HL133560 and NCI R01 CA223194 through Loyola University Chicago and NIAAA R01 AA020518 and U01 AA024733 through Kansas University Medical Center. It was also supported by Loyola Program Development funding to Jiwang Zhang.

Received: September 7, 2021
Revised: April 14, 2022
Accepted: April 14, 2022
Published: May 12, 2022

REFERENCES

Aird, K.M., Worth, A.J., Snyder, N.W., Lee, J.V., Sivanand, S., Liu, Q., Blair, I.A., Wellen, K.E., and Zhang, R. (2015). ATM couples replication stress and metabolic reprogramming during cellular senescence. Cell Rep. *11*, 893–901. https://doi.org/10.1016/j.celrep.2015.04.014.

Boniver, J., Humblet, C., and Defresne, M.P. (1989). Tumor necrosis factor and interferon gamma inhibit the development of radiation-induced thymic lymphomas in C57BL/Ka mice. Leukemia *3*, 611–613.

Brault, M., Olsen, T.M., Martinez, J., Stetson, D.B., and Oberst, A. (2018). Intracellular nucleic acid sensing triggers necroptosis through synergistic type I IFN and TNF signaling. J. Immunol. *200*, 2748–2756. https://doi.org/10.4049/jimmunol.1701492.

Cadet, J., and Davies, K.J.A. (2017). Oxidative DNA damage & repair: an introduction. Free Radic. Biol. Med. *107*, 2–12. https://doi.org/10.1016/j.freeradbiomed.2017.03.030.

Campisi, J., and d'Adda di Fagagna, F. (2007). Cellular senescence: when bad things happen to good cells. Nat. Rev. Mol. Cell Biol. *8*, 729–740. https://doi.org/10.1038/nrm2233.

Carbonneau, C.L., Despars, G., Rojas-Sutterlin, S., Fortin, A., Le, O., Hoang, T., and Beausejour, C.M. (2012). Ionizing radiation-induced expression of INK4a/ARF in murine bone marrow-derived stromal cell populations interferes with bone marrow homeostasis. Blood *119*, 717–726. https://doi.org/10.1182/blood-2011-06-361626.

Domen, J., Gandy, K.L., and Weissman, I.L. (1998). Systemic overexpression of BCL-2 in the hematopoietic system protects transgenic mice from the consequences of lethal irradiation. Blood *91*, 2272–2282. https://doi.org/10.1182/blood.v91.7.2272.2272_2272_2282.

Fessler, E., Eckl, E.M., Schmitt, S., Mancilla, I.A., Meyer-Bender, M.F., Hanf, M., Philippou-Massier, J., Krebs, S., Zischka, H., and Jae, L.T. (2020). A pathway coordinated by DELE1 relays mitochondrial stress to the cytosol. Nature *579*, 433–437. https://doi.org/10.1038/s41586-020-2076-4.

Gutierrez-Martinez, P., Hogdal, L., Nagai, M., Kruta, M., Singh, R., Sarosiek, K., Nussenzweig, A., Beerman, I., Letai, A., and Rossi, D.J. (2018). Diminished apoptotic priming and ATM signalling confer a survival advantage onto aged haematopoietic stem cells in response to DNA damage. Nat. Cell Biol. *20*, 413–421.

Hidalgo San Jose, L., Sunshine, M.J., Dillingham, C.H., Chua, B.A., Kruta, M., Hong, Y., Hatters, D.M., and Signer, R.A.J. (2020). Modest declines in proteome quality impair hematopoietic stem

cell self-renewal. Cell Rep. *30*, 69–80.e6. https://doi.org/10.1016/j.celrep.2019.12.003.

Hishiya, A., Ito, M., Aburatani, H., Motoyama, N., Ikeda, K., and Watanabe, K. (2005). Ataxia telangiectasia mutated (Atm) knockout mice as a model of osteopenia due to impaired bone formation. Bone *37*, 497–503. https://doi.org/10.1016/j.bone.2005.05.012.

Hitosugi, T., Fan, J., Chung, T.W., Lythgoe, K., Wang, X., Xie, J., Ge, Q., Gu, T.L., Polakiewicz, R.D., Roesel, J.L., et al. (2011). Tyrosine phosphorylation of mitochondrial pyruvate dehydrogenase kinase 1 is important for cancer metabolism. Mol. Cell *44*, 864–877. https://doi.org/10.1016/j.molcel.2011.10.015.

Hockendorf, U., Yabal, M., Herold, T., Munkhbaatar, E., Rott, S., Jilg, S., Kauschinger, J., Magnani, G., Reisinger, F., Heuser, M., et al. (2016). RIPK3 restricts myeloid leukemogenesis by promoting cell death and differentiation of leukemia initiating cells. Cancer Cell *30*, 75–91. https://doi.org/10.1016/j.ccell.2016.06.002.

Humblet, C., Greimers, R., Delvenne, P., Deman, J., Boniver, J., and Defresne, M.P. (1996). Prevention of murine radiogenic thymic lymphomas by tumor necrosis factor or by marrow grafting. J. Natl. Cancer Inst. *88*, 824–831. https://doi.org/10.1093/jnci/88.12.824.

Kaiser, W.J., Sridharan, H., Huang, C., Mandal, P., Upton, J.W., Gough, P.J., Sehon, C.A., Marquis, R.W., Bertin, J., and Mocarski, E.S. (2013). Toll-like receptor 3-mediated necrosis via TRIF, RIP3, and MLKL. J. Biol. Chem. *288*, 31268–31279. https://doi.org/10.1074/jbc.m113.462341.

Kang, H.T., Park, J.T., Choi, K., Kim, Y., Choi, H.J.C., Jung, C.W., Lee, Y.S., and Park, S.C. (2017). Chemical screening identifies ATM as a target for alleviating senescence. Nat. Chem. Biol. *13*, 616–623. https://doi.org/10.1038/nchembio.2342.

Kang, S., Fernandes-Alnemri, T., Rogers, C., Mayes, L., Wang, Y., Dillon, C., Roback, L., Kaiser, W., Oberst, A., Sagara, J., et al. (2015). Caspase-8 scaffolding function and MLKL regulate NLRP3 inflammasome activation downstream of TLR3. Nat. Commun. *6*, 7515. https://doi.org/10.1038/ncomms8515.

Kaplon, J., Zheng, L., Meissl, K., Chaneton, B., Selivanov, V.A., Mackay, G., van der Burg, S.H., Verdegaal, E.M.E., Cascante, M., Shlomi, T., et al. (2013). A key role for mitochondrial gatekeeper pyruvate dehydrogenase in oncogene-induced senescence. Nature *498*, 109–112. https://doi.org/10.1038/nature12154.

Kim, J.H., Jenrow, K.A., and Brown, S.L. (2014). Mechanisms of radiation-induced normal tissue toxicity and implications for future clinical trials. Radiat. Oncol. J. *32*, 103–115. https://doi.org/10.3857/roj.2014.32.3.103.

Lee, S.C.W., North, K., Kim, E., Jang, E., Obeng, E., Lu, S.X., Liu, B., Inoue, D., Yoshimi, A., Ki, M., et al. (2018). Synthetic lethal and convergent biological effects of cancer-associated spliceosomal gene mutations. Cancer Cell *34*, 225–241.e8. https://doi.org/10.1016/j.ccell.2018.07.003.

Malumbres, M., Perez de Castro, I., Santos, J., Fernandez Piqueras, J., and Pellicer, A. (1999). Hypermethylation of the cell cycle inhibitor p15INK4b 3'-untranslated region interferes with its transcriptional regulation in primary lymphomas. Oncogene *18*, 385–396. https://doi.org/10.1038/sj.onc.1202299.

Mavragani, I.V., Laskaratou, D.A., Frey, B., Candeias, S.M., Gaipl, U.S., Lumniczky, K., and Georgakilas, A.G. (2016). Key mechanisms involved in ionizing radiation-induced systemic effects. A current review. Toxicol. Res. *5*, 12–33. https://doi.org/10.1039/c5tx00222b.

Milanovic, M., Fan, D.N.Y., Belenki, D., Dabritz, J.H.M., Zhao, Z., Yu, Y., Dorr, J.R., Dimitrova, L., Lenze, D., Monteiro Barbosa, I.A., et al. (2018). Senescence-associated reprogramming promotes cancer stemness. Nature *553*, 96–100. https://doi.org/10.1038/nature25167.

Milyavsky, M., Gan, O.I., Trottier, M., Komosa, M., Tabach, O., Notta, F., Lechman, E., Hermans, K.G., Eppert, K., Konovalova, Z., et al. (2010). A distinctive DNA damage response in human hematopoietic stem cells reveals an apoptosis-independent role for p53 in self-renewal. Cell Stem Cell *7*, 186–197. https://doi.org/10.1016/j.stem.2010.05.016.

Mohrin, M., Bourke, E., Alexander, D., Warr, M.R., Barry-Holson, K., Le Beau, M.M., Morrison, C.G., and Passegue, E. (2010). Hematopoietic stem cell quiescence promotes error-prone DNA repair and mutagenesis. Cell Stem Cell *7*, 174–185. https://doi.org/10.1016/j.stem.2010.06.014.

Moriwaki, K., Balaji, S., McQuade, T., Malhotra, N., Kang, J., and Chan, F.M. (2014). The necroptosis adaptor RIPK3 promotes injury-induced cytokine expression and tissue repair. Immunity *41*, 567–578. https://doi.org/10.1016/j.immuni.2014.09.016.

Mosteiro, L., Pantoja, C., Alcazar, N., Marion, R.M., Chondronasiou, D., Rovira, M., Fernandez-Marcos, P.J., Munoz-Martin, M., Blanco-Aparicio, C., Pastor, J., et al. (2016). Tissue damage and senescence provide critical signals for cellular reprogramming in vivo. Science *354*, aaf4445. https://doi.org/10.1126/science.aaf4445.

Ohnishi, H., Kawamura, M., Ida, K., Sheng, X.M., Hanada, R., Nobori, T., Yamamori, S., and Hayashi, Y. (1995). Homozygous deletions of p16/MTS1 gene are frequent but mutations are infrequent in childhood T-cell acute lymphoblastic leukemia. Blood *86*, 1269–1275. https://doi.org/10.1182/blood.v86.4.1269.bloodjournal8641269.

Palacio, L., Krishnan, V., Le, N.L.O., Sharpless, N.E., and Beausejour, C.M. (2017). Sustained p16(INK4a) expression is required to prevent IR-induced tumorigenesis in mice. Oncogene *36*, 1309–1314. https://doi.org/10.1038/onc.2016.298.

Qiu, X., Zhang, Y., and Han, J. (2018). RIP3 is an upregulator of aerobic metabolism and the enhanced respiration by necrosomal RIP3 feeds back on necrosome to promote necroptosis. Cell Death Differ. *25*, 821–824. https://doi.org/10.1038/s41418-018-0075-x.

Ritschka, B., Storer, M., Mas, A., Heinzmann, F., Ortells, M.C., Morton, J.P., Sansom, O.J., Zender, L., and Keyes, W.M. (2017). The senescence-associated secretory phenotype induces cellular plasticity and tissue regeneration. Genes Dev. *31*, 172–183. https://doi.org/10.1101/gad.290635.116.

Schock, S.N., Chandra, N.V., Sun, Y., Irie, T., Kitagawa, Y., Gotoh, B., Coscoy, L., and Winoto, A. (2017). Induction of necroptotic cell death by viral activation of the RIG-I or STING pathway. Cell Death Differ. *24*, 615–625. https://doi.org/10.1038/cdd.2016.153.

Shao, L., Feng, W., Li, H., Gardner, D., Luo, Y., Wang, Y., Liu, L., Meng, A., Sharpless, N.E., and Zhou, D. (2014). Total body

irradiation causes long-term mouse BM injury via induction of HSC premature senescence in an Ink4a- and Arf-independent manner. Blood *123*, 3105–3115. https://doi.org/10.1182/blood-2013-07-515619.

Shao, L., Sun, Y., Zhang, Z., Feng, W., Gao, Y., Cai, Z., Wang, Z.Z., Look, A.T., and Wu, W.S. (2010). Deletion of proapoptotic Puma selectively protects hematopoietic stem and progenitor cells against high-dose radiation. Blood *115*, 4707–4714. https://doi.org/10.1182/blood-2009-10-248872.

Signer, R.A.J., Magee, J.A., Salic, A., and Morrison, S.J. (2014). Haematopoietic stem cells require a highly regulated protein synthesis rate. Nature *509*, 49–54. https://doi.org/10.1038/nature13035.

Sulong, S., Moorman, A.V., Irving, J.A.E., Strefford, J.C., Konn, Z.J., Case, M.C., Minto, L., Barber, K.E., Parker, H., Wright, S.L., et al. (2009). A comprehensive analysis of the CDKN2A gene in childhood acute lymphoblastic leukemia reveals genomic deletion, copy number neutral loss of heterozygosity, and association with specific cytogenetic subgroups. Blood *113*, 100–107. https://doi.org/10.1182/blood-2008-07-166801.

Tang, M., Wei, X., Guo, Y., Breslin, P., Zhang, S., Zhang, S., Wei, W., Xia, Z., Diaz, M., Akira, S., and Zhang, J. (2008). TAK1 is required for the survival of hematopoietic cells and hepatocytes in mice. J. Exp. Med. *205*, 1611–1619. https://doi.org/10.1084/jem.20080297.

Upton, J.W., Kaiser, W.J., and Mocarski, E.S. (2012). DAI/ZBP1/DLM-1 complexes with RIP3 to mediate virus-induced programmed necrosis that is targeted by murine cytomegalovirus vIRA. Cell Host and Microbe *11*, 290–297. https://doi.org/10.1016/j.chom.2012.01.016.

van Galen, P., Mbong, N., Kreso, A., Schoof, E.M., Wagenblast, E., Ng, S.W.K., Krivdova, G., Jin, L., Nakauchi, H., and Dick, J.E. (2018). Integrated stress response activity marks stem cells in normal hematopoiesis and leukemia. Cell Rep. *25*, 1109–1117.e5. https://doi.org/10.1016/j.celrep.2018.10.021.

Van Herreweghe, F., Festjens, N., Declercq, W., and Vandenabeele, P. (2010). Tumor necrosis factor-mediated cell death: to break or to burst, that's the question. Cell Mol. Life Sci. *67*, 1567–1579. https://doi.org/10.1007/s00018-010-0283-0.

Wagner, P.N., Shi, Q., Salisbury-Ruf, C.T., Zou, J., Savona, M.R., Fedoriw, Y., and Zinkel, S.S. (2019). Increased Ripk1-mediated bone marrow necroptosis leads to myelodysplasia and bone marrow failure in mice. Blood *133*, 107–120. https://doi.org/10.1182/blood-2018-05-847335.

Wang, S., Ni, H.M., Dorko, K., Kumer, S.C., Schmitt, T.M., Nawabi, A., Komatsu, M., Huang, H., and Ding, W.X. (2016). Increased hepatic receptor interacting protein kinase 3 expression due to impaired proteasomal functions contributes to alcohol-induced steatosis and liver injury. Oncotarget *7*, 17681–17698. https://doi.org/10.18632/oncotarget.6893.

Wang, X., Jiang, W., Yan, Y., Gong, T., Han, J., Tian, Z., and Zhou, R. (2014). RNA viruses promote activation of the NLRP3 inflammasome through a RIP1-RIP3-DRP1 signaling pathway. Nat. Immunol. *15*, 1126–1133. https://doi.org/10.1038/ni.3015.

Wang, Y., Liu, L., Pazhanisamy, S.K., Li, H., Meng, A., and Zhou, D. (2010). Total body irradiation causes residual bone marrow injury by induction of persistent oxidative stress in murine hematopoietic stem cells. Free Radic. Biol. Med. *48*, 348–356. https://doi.org/10.1016/j.freeradbiomed.2009.11.005.

Wang, Y., Liu, L., and Zhou, D. (2011). Inhibition of p38 MAPK attenuates ionizing radiation-induced hematopoietic cell senescence and residual bone marrow injury. Radiat. Res. *176*, 743–752. https://doi.org/10.1667/rr2727.1.

Wang, Y., Schulte, B.A., LaRue, A.C., Ogawa, M., and Zhou, D. (2006). Total body irradiation selectively induces murine hematopoietic stem cell senescence. Blood *107*, 358–366. https://doi.org/10.1182/blood-2005-04-1418.

Xiao, Y., Li, H., Zhang, J., Volk, A., Zhang, S., Wei, W., Zhang, S., Breslin, P., and Zhang, J. (2011). TNF-α/Fas-RIP-1–induced cell death signaling separates murine hematopoietic stem cells/progenitors into 2 distinct populations. Blood *118*, 6057–6067. https://doi.org/10.1182/blood-2011-06-359448.

Xin, J., Breslin, P., Wei, W., Li, J., Gutierrez, R., Cannova, J., Ni, A., Ng, G., Schmidt, R., Chen, H., et al. (2016a). Necroptosis in spontaneously-mutated hematopoietic cells induces autoimmune bone marrow failure in mice. Haematologica *102*, 295–307.

Xin, J., You, D., Breslin, P., Li, J., Zhang, J., Wei, W., Cannova, J., Volk, A., Gutierrez, R., Xiao, Y., et al. (2016b). Sensitizing acute myeloid leukemia cells to induced differentiation by inhibiting the RIP1/RIP3 pathway. Leukemia *31*, 1154–1165. https://doi.org/10.1038/leu.2016.287.

Yamashita, M., and Passegue, E. (2019). TNF-alpha coordinates hematopoietic stem cell survival and myeloid regeneration. Cell Stem Cell *25*, 357–372.e7. https://doi.org/10.1016/j.stem.2019.05.019.

Yang, Z., Wang, Y., Zhang, Y., He, X., Zhong, C.Q., Ni, H., Chen, X., Liang, Y., Wu, J., Zhao, S., et al. (2018a). RIP3 targets pyruvate dehydrogenase complex to increase aerobic respiration in TNF-induced necroptosis. Nat. Cell Biol. *20*, 186–197. https://doi.org/10.1038/s41556-017-0022-y.

Yang, Z., Wang, Y., Zhang, Y., He, X., Zhong, C.Q., Ni, H., Chen, X., Liang, Y., Wu, J., Zhao, S., et al. (2018b). RIP3 targets pyruvate dehydrogenase complex to increase aerobic respiration in TNF-induced necroptosis. Nat. Cell Biol. *20*, 186–197. https://doi.org/10.1038/s41556-017-0022-y.

Stem Cell Reports, Volume *17*

Supplemental Information

Ripk3 signaling regulates HSCs during stress and represses radiation-induced leukemia in mice

Lei Zhang, Huacheng Luo, Hong-Min Ni, Shanhui Liu, Hongyun Xing, Jun Zhang, Mark Sellin, Peter Breslin, S.J., Wei Wei, Mitchell F. Denning, William Small Jr., Wen-Xing Ding, Suming Huang, and Jiwang Zhang

Supplemental figures

Figure S1. Strategy for analysis of BM HSCs and HPCs.

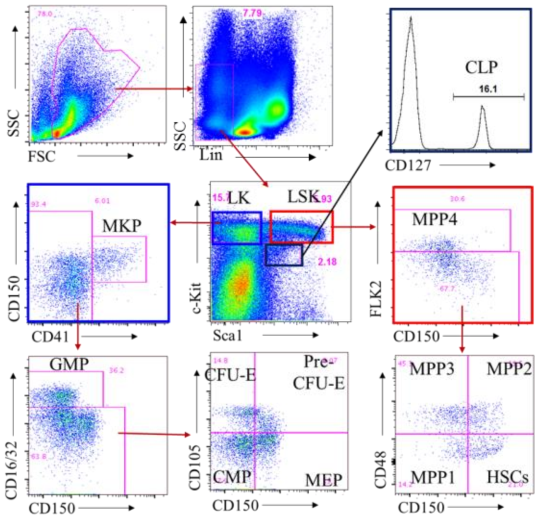

LSK populations:
CD150^{+}FLk2^{-}CD48^{-} HSCs,
CD150^{-}FLk2^{-}CD48^{-} MPP1,
CD150^{+}FLk2^{-}CD48^{+} MPP2,
CD150^{-}FLk2^{-}CD48^{+} MPP3
CD150^{-}FLk2^{+}CD48^{+} MPP4.

LK-myeloid progenitors (LK-MPs):
CD34^{+}CD16/32^{-}CD150^{-}CD105^{-} common myeloid progenitors (CMP),
CD16/32^{+} granulocyte/monocyte progenitors (GMP),
CD16/32^{-}CD150^{+}CD105^{-} megakaryocyte/erythrocyte progenitors (MEP),
CD150^{+}CD41^{+} megakaryocyte progenitors (MKP),
CD16/32^{-}CD150^{+}CD105^{+} pre-colony forming unit-erythroid (pre-CFU-E),
CD16/32^{-}CD150^{-}CD105^{+} colony forming unit-erythroid (CFU-E)

Lin^{-}Sca1^{low}c-kit^{low}CD127^{+} common lymphocyte progenitors (CLP)

Figure S2. Normal BM hematopoiesis in Ripk3$^{-/-}$ and Mlkl$^{-/-}$ mice under normal homeostatic conditions. (Associated with Figure 1) BM were collected from *WT*, *Ripk3*$^{-/-}$ and *Mlkl*$^{-/-}$ mice. Six mice per genotype were studied (3 males, 3 females). All mice were analyzed at 6 months of age. Percentages of LK, LSK, HSCs and MPPs (**a**), and percentages of MKP, CMP, GMP, MEP, pre-CFU-E, CFU-E, and CLP (**b**) in the BM of *WT*, *Ripk3*$^{-/-}$ and *Mlkl*$^{-/-}$ mice are shown.

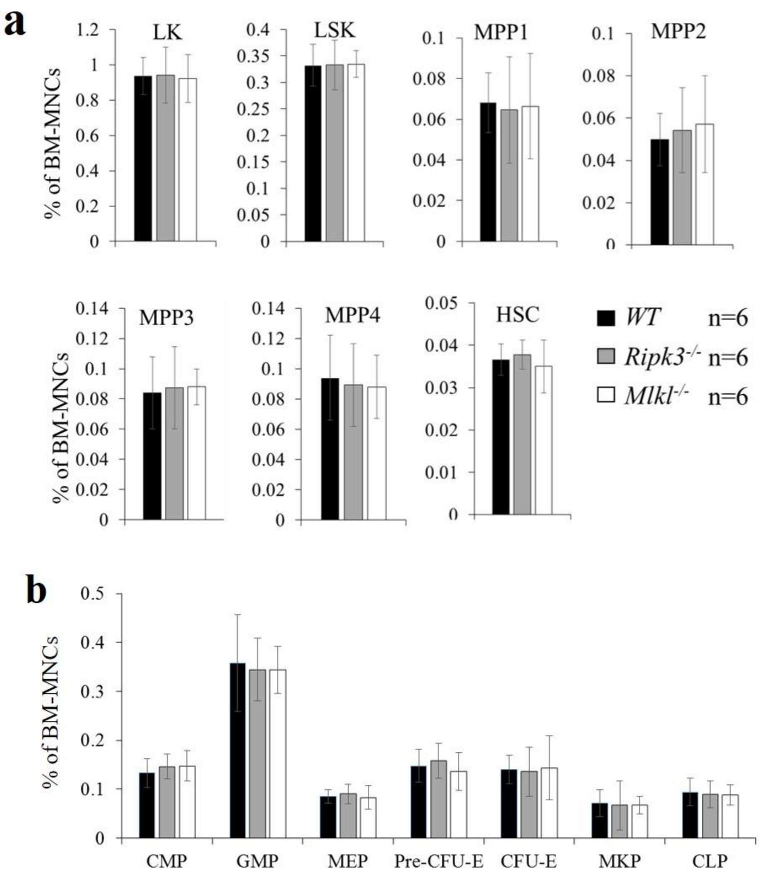

Figure S3. Ripk3 selectively regulates number and function of HSCs during serial transplantation in Mlkl-dependent and -independent manners, respectively. (Associated with Figure 2) **a-c**. BM MNCs were collected from 3rd transplantation recipients of the indicated genotypes of donors. Percentages of LK cells and LSK cells (**a**), as well as HSCs and MPPs (**b**) in BM from 3rd transplantation recipients of the indicated genotypes of donors are shown. The percentages of $CD11b^{+}$ granulocytes/monocytes, $B220^{+}$ B lymphocytes and $CD3^{+}$ T lymphocytes in PB were analyzed by flow cytometry (**c**). **d**. BM MNCs were collected from *WT*, *Ripk3*$^{-/-}$ and *Mlkl*$^{-/-}$ mice (all were $CD45.2^{+}$) and mixed with equal numbers of competitor BM MNCs ($CD45.1^{+}$), respectively. The mixture of cells was studied using serial transplantation assay. The CHRC of the donor BM MNCs was examined by analyzing the $CD45.2^{+}$ cell % in the PB of the recipients after 4 months of each cycle of transplantation. * and & indicate $p<0.05$ when compared to *WT* and *Ripk3*$^{-/-}$ groups, respectively.

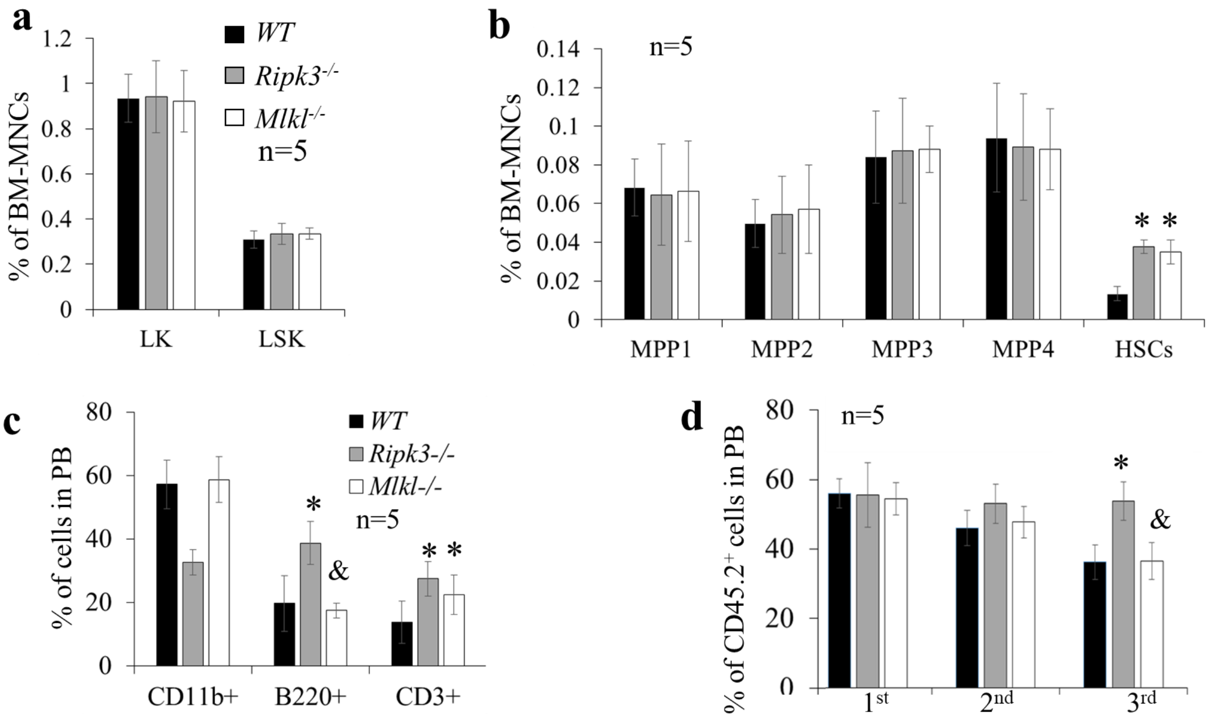

Figure S4. Ripk3 depletion prevents low dose radiation-induced loss of HSCs. (Associated with Figure 4) **a-b.** *WT, Ripk3*$^{-/-}$ and *Mlkl*$^{-/-}$ mice were irradiated with X-rays, 1.75Gy weekly × 4. BM MNCs were collected from mice 1 month following the last IR. Percentages of HSCs and HPCs (**a**) and the numbers of MNCs (**b**) in the BM of indicated genotypes of mice are presented. Five mice were studied in each group. * and ** stand for p<0.05 and p<0.01, respectively, when compared to non-irradiated *WT* mice. $ indicates p<0.05 when compared irradiated *Ripk3*$^{-/-}$ and *Mlkl*$^{-/-}$ mice. **c.** *WT* and *Ripk3*$^{-/-}$ mice were irradiated with 1.75Gy X-rays every week. BM MNCs were collected from mice 14 and 21 days following the first IR. HSCs and HPCs were analyzed by flow cytometry. Representative flow cytometric data are presented. & indicates p<0.05 when compared to irradiated *Ripk3*$^{-/-}$ mice. **d.** Lethally-irradiated mice (CD45.1^{+}) were transplanted with a mixture of *WT* (CD45.1^{+}) and *Ripk3*$^{-/-}$ (CD45.2^{+}) BM MNCs at a 1:1 ratio. One month after transplantation, mice were irradiated with 1.75Gy X-rays every week ×4 weeks. BM MNCs were collected 1 week following the last IR. The numbers of *WT* and *Ripk3*$^{-/-}$ HSCs and MPPs were analyzed by flow cytometry based on CD45.1 and CD45.2 expression. ** indicates p<0.01 compared to *WT* mice. **e.** *WT* mice were irradiated with 1.75Gy X-rays weekly. BM MNCs were collected from mice 14 and 30 days following the first IR. LSK-CD150^{+}CD48^{-}HSCs were purified by FACS and p-Ripk3 levels were analyzed by intracellular antibody staining. Data show one of the 3 biological triplicate experiments.

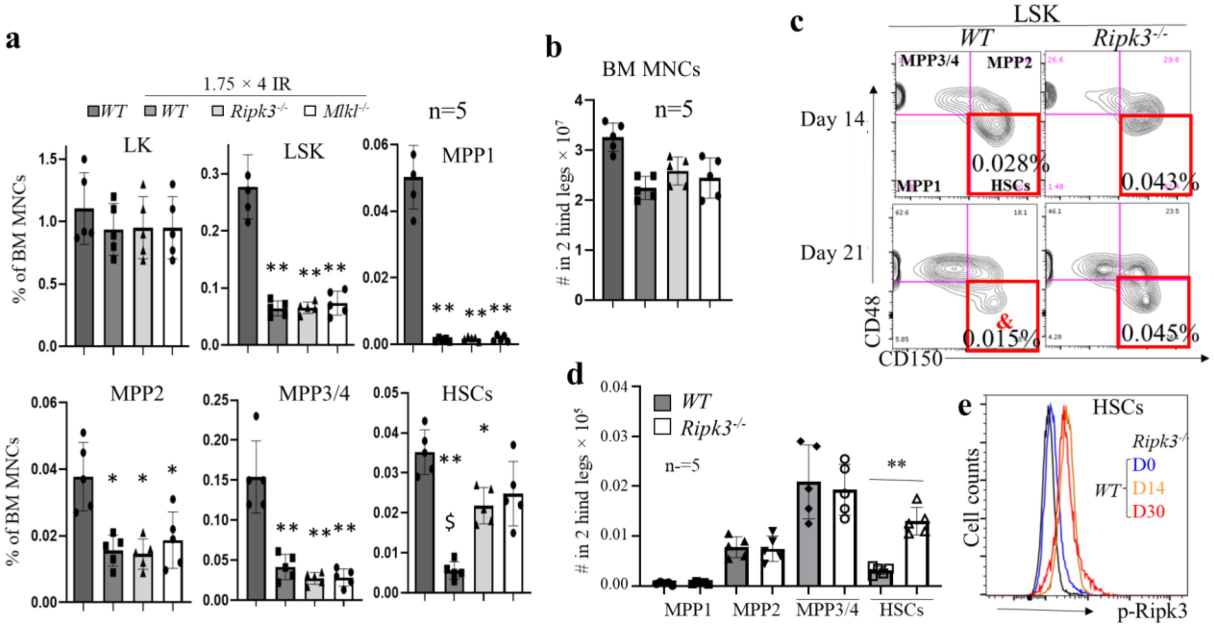

Figure S5. Depletion of either Tnfr or Ripk3 prevents low dose radiation-induced loss of HSCs. (Associated with Figure 4) **a-b.** *WT*, *Tnfr$^{-/-}$* and *Ripk3$^{-/-}$* mice were irradiated with 1.75Gy X-rays every week ×4 weeks. BM MNCs were collected 1 month following the last IR. p-Ripk3 levels (**a**) in LSK cells and numbers of HSCs (**b**) were analyzed by flow cytometry. Data in **a** show one of the 3 biological triplicate experiments. ** indicates for p<0.01 compared to *WT* mice.

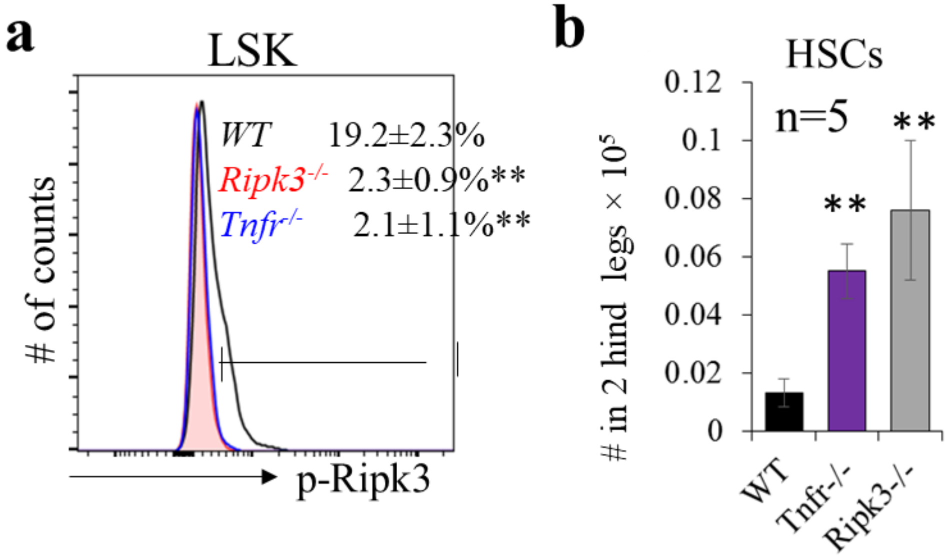

Figure S6. Tnf-Ripk3 signaling did not alter mitochondrial-ISR in LK HPCs. (Associated with Figure 6) *WT*, *Ripk3$^{-/-}$*, *Mlkl$^{-/-}$* and *Tnfr$^{-/-}$* mice were irradiated with X-ray, 1.75Gy weekly ×4. LK populations were collected from mouse BM 1 month after the last IR. p-eIF2a levels were examined by flow cytometry. Data in **a** and **c** show one of the 3 biological triplicate experiments.

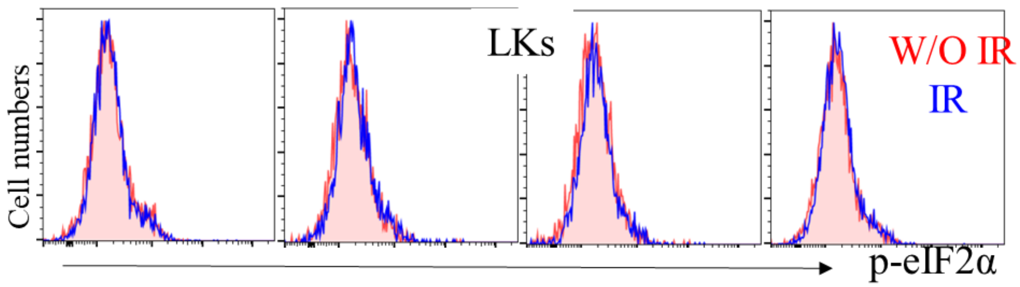

Figure S7. Inhibition of PDH or ROS restores eIF2α-Atf4-ISR signaling in radiated Mlkl $^{-/-}$ ***HSCs.*** (Associated with Figure 7) *Mlkl* $^{-/-}$ mice were irradiated with X-ray, 1.75Gy weekly × 4 and treated with vehicle (Veh), CPI-613 or NAC, respectively. LSK cells were collected from mouse BM 1 month after the last IR. LSKs isolated from IR *Ripk3* $^{-/-}$ mice were studied as controls. mtROS (**a**), p-eIF2a (**b**), protein synthesis rate (**c**), and senescence (**d**) were examined by flow cytometry. Data in **a-d** show one of the 3 biological triplicate experiments.

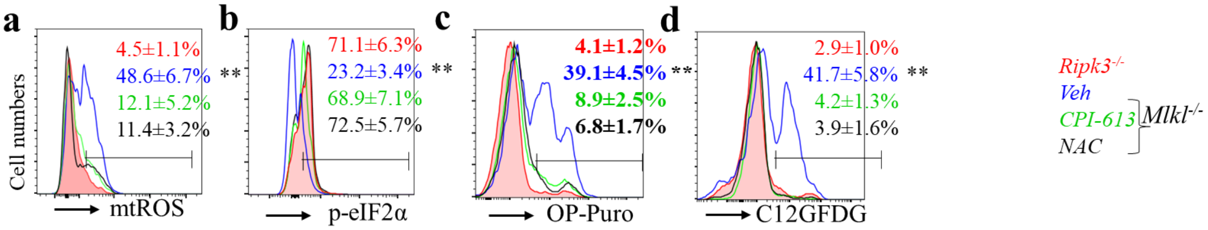

Table 1. Primers for mouse genotypic analysis:

PCR primer sequences for genotyping of *Ripk3*$^{-/-}$ mice
Ripk3-f: 5'-ggctttcattgtggaggtaagctgaga-3';
Ripk3-r: 5'-gaacccgtggataagtgcacttgaat-3'.
These primers generate a 280bp product in *WT* and a 320bp product in *Ripk3*-mutant mice.
PCR primer sequences for genotyping of *Mlkl*$^{-/-}$ mice:
Mlkl-p1: 5'-tatgaccatggcaactcacg-3',
Mlkl-p2: 5'-accatctccccaaactgtga-3'
Mlkl-p3: 5'-tccttccagcacctcgta at-3'.
These primers generate a 498bp *WT* product and a 158bp product for the recombined Δ-exon 3 *Mlkl* gene.
PCR primer sequences for genotyping of *Tnfr*$^{-/-}$ mice:
Tnfr1a-p1: 5'-ggattgtcacggtgccgttgaag-3'
Tnfr1a-p2: 5'-tgacaaggacacggtgtgtggc-3'
Tnfr1a-p3: 5'-tgctgatggggatacatccatc-3'
These primers generate a 120bp *WT* product and a 155bp product in *Tnfr1a*-mutant mice.

Table 2. Primers used for qRT-PCR:

Taqman primers and probes	Purchased from Thermo Fisher Scientific
Bcl2	*Mm00477631_m1*
cIAP2	*Mm01168413_m1*
Bcl-x	*Mm00437783_m1*
Puma	*Mm00519268_m1*
NoxA	*Mm00451763_m1*
Bim	*Mm00437783_m1*
p16	*Mm00494449_m1*
p19	*Mm00486943_m1*
p15	*Mm00483241_m1*
p53	*Mm01731290_g1*
Gapdh	*Mm99999915_g1*
Calcrl	*Mm00516985_m1*
Klf4	*Mm00516104_m1*
Wbp4	*Mm01324202_m1*
Fkbp1a	*Mm01243847_g1*
P2ry13	*Mm00546978_m1*
Banp	*Mm00479456_m1*
Lcn2	*Mm01324470_m1*
Snx20	*Mm00446029_m1*
Primers	for qRT-PCR using SYBR Green assay
Atf4-f	5′-aacaagacagcagccactaggt,
Atf4-r	5′-tctgccttctctttcagagcctca
Grp78-f	5′-cagatcttctccacggct-3′
Grp78-r	5′-tgtcactcggagaatacc at-3′
Chop-f	5′-ccagaataacagccggaacctga-3′
Chop-r	5′-tcctgcagatcctcataccag-3′;
Erdj4-f	5′-ggatggttctagtagacaaagg-3′
Erdj4-r	5′-cttcgttgagtgacagtcctgc-3′
Dnajb8-f	5′-tcggtgctggtcatcccctt-3′
Dnajb8-r	5′-gggaactcaccaaagcccgaa-3′
Xbp1s-f	5′-gagtccgcagcaggtg-3′
Xbp1s-r	5′-gtgtcagagtccatggga-3′

Xbp1 total-f	5′-aaacagagtagcagcgcagac-3′
Xbp1 total-r	5′-caggatccagcgtgtcca t-3′
β-actin-f	5′-ggctgtattcccctccatcg-3′
β-actin-r	5′-ccagttggtaacaatgccatg t-3′
RT-PCR primers	for detecting xbp1 splicing:
xbp1 splicing-f	5′-acacgcttgggaatggaac-3′
xbp1 splicing-r	5′-ccatgggaagatgttctggg-3′

Table 3. Antibodies for flow cytometric analysis and Western blot analysis

**Antibodies for intracellular staining and flow cytometry**	Venders	Cat #
Phospho-p38 MAPK (Thr180/Tyr182) (3D7) Rabbit mAb (Alexa Fluor® 647 Conjugate)	Cell Signaling Technology	14594
Phospho-NF-κB p65 (Ser536) (93H1) Rabbit mAb (Alexa Fluor® 647 Conjugate)	Cell Signaling Technology	4887
Phospho-RIP3 (Thr231/Ser232) (E7S1R) Rabbit mAb (Alexa Fluor® 488 Conjugate)	Cell Signaling Technology	47477
p44/42 MAPK (Erk1/2) (137F5) Rabbit mAb (Alexa Fluor® 647 Conjugate)	Cell Signaling Technology	5376
Phospho-GSK-3β (Ser9) (D85E12) XP® Rabbit mAb (Alexa Fluor® 647 Conjugate)	Cell Signaling Technology	14332
Phospho-eIF2α (Ser51) (D9G8) XP® Rabbit mAb (Biotinylated)	Cell Signaling Technology	5199
Alexa Fluor 488 Goat anti-Rabbit IgG	ThermoFisher Scientific	A-11034
Rabbit monoclonal phospho-eIF2a (Ser51)	ThermoFisher Scientific	44-728G
FAM-FLICA® Caspase-1 (YVAD) Assay Kit	ImmunoChemistry Technologies	97
**Antibodies for Western blotting**		
RIP3 (D4G2A) Rabbit mAb (Mouse-Specific)	Cell Signaling Technology	95702
Anti-RIP3 antibody (Mouse-Specific)	Abcam	ab56164
Phospho-RIP3 (Thr231/Ser232) Antibody (Mouse-Specific)	Cell Signaling Technology	57220
Phospho-MLKL (Ser345) (D6E3G) Rabbit mAb	Cell Signaling Technology	37333
MLKL (D6W1K) Rabbit mAb (Mouse-Specific)	Cell Signaling Technology	37705
OPA1 (D6U6N) Rabbit mAb	Cell Signaling Technology	80471
ATF-4 (D4B8) Rabbit mAb	Cell Signaling Technology	11815
Phospho-PERK (Thr980) Monoclonal Antibody (G.305.4)	Thermofisher Scientific	MA5-15033
**Antibodies for HSCs and HPCs analysis**		
Brilliant Violet 421™ anti-mouse Ly-6G/Ly-6C (Gr-1) Antibody	Biolegend	108434
Brilliant Violet 421™ anti-mouse CD3ε Antibody	Biolegend	100336
Brilliant Violet 421™ anti-mouse B220 Antibody	Biolegend	103240
Brilliant Violet 421™ anti-mouse TER-119/Erythroid Cells Antibody	Biolegend	116233
PE anti-mouse Ly-6A/E (Sca-1) Antibody	Biolegend	108108
APC anti-mouse CD117 (c-Kit) Antibody	Biolegend	105812
APC/Cy7 anti-mouse CD48 Antibody	Biolegend	103431
PE/Cy7 anti-mouse CD150 (SLAM) Antibody	Biolegend	115914
PE/Cy5 anti-mouse CD135 Antibody	Biolegend	135312
PerCP/Cy5.5 anti-mouse CD127 (IL-7Rα) Antibody	Biolegend	135022
CD41a Monoclonal Antibody (eBioMWReg30 (MWReg30)), PerCP-eFluor 710	Life technologies	46-0411
FITC anti-mouse CD117	BD Bioscience	553354
PE-Cy7 anti-mouse CD3e	BD Bioscience	552774
PE anti-mouse CD11b	Thermo Fisher Scientific	12-0112-85
APC Anti-human/mouse CD45R	Thermo Fisher Scientific	17-0452-82
PE anti-mouse CD8a	Thermo Fisher Scientific	12-0081-82
APC Anti-mouse CD4	Thermo Fisher Scientific	17-0041-83
FITC CD3e Monoclonal Antibody (145-2C11),	Thermo Fisher Scientific	11-0031-82

Supplementary Material and Methods

Analysis of levels of intracellular ROS and mitochondrial ROS. BM-MNCs (1×10^6/ml) were stained with antibodies against lineage markers, anti-Sca-1-PE/FITC and anti-c-kit-APC antibodies in PBS supplemented with 5mM glucose, 1mM $CaCl_2$, 0.5mM $MgSO_4$, and 5mg/ml BSA and then incubated with 10µM of the cell-permeant 2',7'-dichlorodihydrofluorescein diacetate (H2DCFDA) (ThermoFisher, D399) or MitoSOX™ Red Mitochondrial Superoxide Indicator (ThermoFisher, M36008) for 10-30 min. at 37 ℃. Levels of intracellular ROS and mitochondrial ROS in LK- and LSK-cells were analyzed using a FACS Fortessa flow cytometer (Becton-Dickinson, San Jose, CA, USA). For each sample, a minimum of 300,000 BM-MNCs were acquired and the data were analyzed using Flowjo software. In all experiments, PE and APC isotype controls and other positive and negative controls were included as appropriate.

MitoTracker analysis: Bone marrow cells were incubated in 20nM MitoTracker Green (M7514; Molecular Probes) according to the manufacturer's instructions for 15 min. at 37 ℃. After washing the cells twice with PBS, cells were stained with HSC cell surface markers. Cells were washed twice with PBS and then analyzed using FACS.

Senescence analysis. SA-β-gal activity in sorted LSK cells was determined using a Senescence β-Galactosidase Staining Kit from Cell Signaling Technology (#9860) according to the manufacturer's instructions and analyzed by flow cytometry.

Seahorse Assays. XF96 extracellular flux assay kits (Agilent Technologies, 102417-100) were used to measure oxygen consumption (OCR). LSK cells were sorted, and directly plated into XF96 cell culture microplates. OCR was measured according to the protocol supplied by the manufacturer. Briefly, five replicate wells of 1-2 $\times 10^5$ cells were seeded into a Cell-Tak coated 96-well XF96 well plate (Corning, 324240). Thirty minutes prior to analysis, the medium was replaced with Seahorse XF medium (Agilent Technologies, 102353-100) and the plate was incubated at 37°C. Analyses were performed at basal conditions.

Measuring protein synthesis. BM MNCs were collected from mice 1 hour after intraperitoneal administration of OP-Puro 50mg/kg body weight (Opropargyl-puromycin; pH 6.4-6.6 in PBS). BM MNCs (4×10^6) were stained with antibodies against cell surface markers as described above. After washing, cells were fixed, permeabilized, and the azide-alkyne cycloaddition was performed using the Click-iT Cell Reaction Buffer Kit (Life Technologies, Cat#: C10269) with 5µM Alexa Fluor™ 488 Azide (Life Technologies). The protein synthesis rate was measured by flow cytometry.

Analysis of apoptosis using Annexin-V staining and caspase activity. LK and LSK cells were stained with propidium iodide and APC-conjugated Annexin-V in Annexin binding buffer following the manufacturer's instructions (BD Biosciences). Apoptosis was further verified by examining Caspase 3 activity using active-Caspase-3 staining kits following the manufacturer's instructions (eBioscience).

Colony-forming cell (CFC) assay. The CFC assay was performed by culturing BM-MNCs in MethoCult GF M3434 methylcellulose medium (Stem Cell Technologies, Vancouver, BC). Colonies of CFU-granulocyte macrophage (GM) and burst-forming unit-erythroid (BFU-E) were

scored on day 7 and those of CFU-granulocyte, -erythrocyte, -monocyte, and -megakaryocyte (GEMM) were scored on day 12 of incubation according to the manufacturer's protocol.

Western blotting. Cell lysates from BM MNCs were extracted using Cell Lysis Buffer (Cell Signaling) followed by a brief sonication. The supernatants were collected after centrifugation at 4 ℃., 14,000 ×g for 20 min. Protein samples were separated by SDS-PAGE on 10% or 12% acrylamide gels. Proteins were transferred onto nitrocellulose membranes to examine the target proteins by antibody blotting. Antibodies used in this study are listed in Antibodies for Western blotting (**Table 3**). The band quantifications using Multi Gauge 3.0 were normalized to corresponding controls, which were set to 1.00 after being normalized to corresponding loading standards on the Western blot.

Serial transplantation assay. BM-MNCs were collected from 2 month-old *WT*, *Ripk3$^{-/-}$* and *Mlkl$^{-/-}$* mice (CD45.2^{+}) and were transplanted into lethally-irradiated (9.5Gy IR) Ptprc (CD45.1^{+}) mice. Five recipients were used in each group, 5 × 10^{6} MNCs were transplanted per recipient. PB and BM were collected from recipient mice 4 months post-transplantation for phenotypic analysis using flow cytometry. MNCs from all 5 recipients in each group were pooled and transplanted into lethally-irradiated 2nd Ptprc recipients, respectively. Five recipients were used in each group, 1 × 10^{7} MNCs were transplanted per recipient. The same procedure was followed for the 3rd transplantation.

Competitive repopulation and serial transplantation assay. Donor BM-MNCs were collected from 2 month-old *WT, Ripk3$^{-/-}$* and *Mlkl$^{-/-}$* mice (CD45.2^{+}) and were mixed, respectively, with equal numbers of competitor BM-NMCs collected from 2-month-old Ptprc (CD45.1^{+}) mice. The mixtures of BM cells were transplanted into lethally-irradiated (9.5Gy) Ptprc (CD45.1^{+}) mice by tail-vein injection. Five recipient mice were used in each group, 5 × 10^{5} donor NMCs and 5 × 10^{5} competitor NMCs per recipient. PB samples were collected from all recipients 4 months after transplantation. Donor cell engraftments were analyzed by examining % of CD45.2^{+} cells in PB as assessed by flow cytometry. T-cell, B-cell, granulocyte, and monocyte/macrophage lineage contributions of the donor cells were analyzed by staining with anti-CD3, anti-B220, anti-Gr-1, and anti-Mac-1, respectively. For 2nd transplantations, MNCs from all 5 recipients in each group were pooled and transplanted into lethally-irradiated 2nd Ptprc recipients, respectively. Five recipients were used per group, 1 × 10^{7} MNCs per recipient. The exact same procedures were followed for the 3rd transplantation.

RNAseq Analysis. LSK and LK cells were purified from BM of *WT* and *Ripk3$^{-/-}$* mice by FACS 1 month after 1.75 × 4 IR. Two biological replicates were analyzed for each sample. Each sample consisted of a mixture of the indicated cells from 4-5 mice. Total RNA was isolated from these cells using TRIzol reagent (Invitrogen, Carlsbad, CA) following the manufacturer's protocol. Raw data FASTQ files were aligned to the mouse genome (mm9) using Tophat (version 2.0) and Bowtie2.(Langmead et al., 2009) TopHat software was used to analyze RNA-Seq data in order to discover splice junctions.(Bai et al., 2016) Gene expression profiles for the individual samples were calculated as FPKM (paired-end fragments per kilobase of exon model per million mapped reads) values. The Gene Ontology analysis was carried out with the Database for Annotation, Visualization and Integrated Discovery (DAVID) tool (https://david.ncifcrf.gov/, Version 6.8).(Huang da et al., 2009) The differential expression cluster for heatmap was generated using

JavaTreeview.(Saldanha, 2004) The RNAseq dataset was deposited into the Gene Expression Omnibus archive (accession no. GSE70899) and GEO number (GSE139143).

Statistical analyses. Data are expressed as means ± SD. Two-way ANOVA (multiple groups) and Student's *t*-test (two groups) were performed to determine the statistical significance of differences among and between experimental groups. $P<0.05$ was considered significant. All these analyses were done using GraphPad Prism from GraphPad Software (San Diego, CA).

References

Bai, Y., Kinne, J., Donham, B., Jiang, F., Ding, L., Hassler, J.R., and Kaufman, R.J. (2016). Read-Split-Run: an improved bioinformatics pipeline for identification of genome-wide non-canonical spliced regions using RNA-Seq data. BMC genomics *17 Suppl 7*, 503.
Huang da, W., Sherman, B.T., and Lempicki, R.A. (2009). Systematic and integrative analysis of large gene lists using DAVID bioinformatics resources. Nature protocols *4*, 44-57.
Langmead, B., Trapnell, C., Pop, M., and Salzberg, S.L. (2009). Ultrafast and memory-efficient alignment of short DNA sequences to the human genome. Genome Biol *10*, R25.
Saldanha, A.J. (2004). Java Treeview--extensible visualization of microarray data. Bioinformatics *20*, 3246-3248.
